# Treatment Trade‐Offs and Choices for Femoral Fractures: A Systematic Review and Meta‐Analysis

**DOI:** 10.1111/os.70001

**Published:** 2025-03-25

**Authors:** Jiarui Li, Zhu Guo, Tianrui Wang, Kunyue Xing, Wenzhuo Wang, Yaowei Liu, Jiyao Xing, Hongfei Xiang, Jingdong Wang, Bohua Chen, Dongming Xing, Xiaolin Wu

**Affiliations:** ^1^ The Affiliated Hospital of Qingdao University, Qingdao Cancer Institute Qingdao University Qingdao China; ^2^ The Affiliated Hospital of Qingdao University, Orthopaedic Hospital Qingdao University Qingdao China; ^3^ Institute of Health Informatics, Faculty of Population Health Sciences University College London London UK; ^4^ Dezhou Municipal Hospital, Department of Orthopedics Dezhou University Dezhou China; ^5^ School of Life Science Tsinghua University Beijing China

**Keywords:** cost‐effectiveness, elderly patients with surgical contraindications, femoral fracture treatment, resource‐limited settings, surgical intervention, traction therapy, treatment decision‐making

## Abstract

In resource‐limited settings, selecting the appropriate treatment for femoral fractures is crucial as it affects both patient recovery and the efficient use of medical resources. This review explores the treatment options for adult and elderly patients with surgical contraindications suffering from femoral fractures, with a particular emphasis on the trade‐offs between surgical intervention and traction therapy. Through a systematic literature search of major databases such as PubMed, Web of Science, and the Cochrane Library, we identified 39 studies that met the inclusion criteria, focusing on complications, treatment effectiveness, functional recovery, and cost analysis. We found that although intramedullary nailing may offer better clinical outcomes, traction therapy often becomes the treatment of choice in resource‐poor environments due to limited surgical resources. The professional judgment of physicians (OR 10.81; 95% CI 8.28–14.11), patient preferences (OR 1.33; 95% CI 0.80–2.21), and hospital surgical capacity (OR 1.87; 95% CI 0.56–6.28) are key factors influencing treatment choice. For elderly patients, the choice of treatment requires a balance between the risks of surgery and the potential complications of non‐surgical treatment (OR 0.78; 95% CI 0.10–5.90). Ultimately, the decision‐making process is complex and requires a comprehensive consideration of available resources, cost‐effectiveness, patient health status, physician experience, patient preferences, and expected clinical outcomes. In resource‐constrained areas, this process is particularly challenging and necessitates a careful consideration of the risks and benefits of both surgical and non‐surgical treatment options.

## Graphical abstract

This review examines the trade‐offs between surgical intervention and traction therapy for adult and elderly patients with femoral fractures and surgical contraindications, particularly in resource‐limited settings. While intramedullary nailing may offer better clinical outcomes, traction therapy is often preferred due to limited surgical resources. Physician judgment, patient preferences, and hospital surgical capacity significantly influence treatment choices. For elderly patients, treatment selection requires balancing the risks of surgery with the potential complications of non‐surgical treatments.

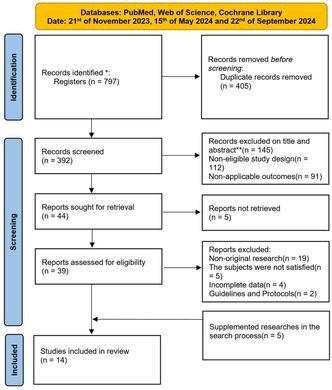

## 1 Introduction

Femoral fractures, as severe traumatic events, have a significant long‐term impact on the quality of life and health status of patients. This is particularly true in the elderly population [1, 2, 3, 4, 5], where the incidence of femoral fractures has significantly increased due to the prevalence of osteoporosis [6, 7, 8] and an elevated risk of falls [9, 10, 11, 12, 13]. Additionally, resource‐limited environments, such as some underdeveloped areas [14, 15], present additional challenges in the selection of treatment modalities. Against this backdrop, choosing the most appropriate treatment method becomes especially crucial.

Surgical treatments, such as intramedullary nailing (IMN) [16] and hip arthroplasty [17, 18, 19], often yield better clinical outcomes but require higher medical resources and economic support. In the study by Bano et al. [20], during hospitalization, there are typically two treatment approaches. One is the conventional orthopedic treatment model, and the other is the interdisciplinary orthogeriatric treatment model. Both approaches involve surgical procedures, but the traditional model may be primarily managed by orthopedic surgeons, whereas the interdisciplinary model involves a collaborative effort between orthopedic surgeons and geriatricians. In contrast, bone traction, as a less costly and less equipment‐demanding treatment method, is frequently considered in resource‐limited settings. However, traction therapy may necessitate a longer recovery time and could potentially lead to more complications [21].

This review aims to explore the selection of treatment methods for femoral fractures in adult patients and elderly patients with surgical contraindications, especially under resource‐limited conditions, focusing on the advantages and disadvantages of surgery versus traction therapy. This study will evaluate the cost‐effectiveness, clinical efficacy, quality of patient recovery, and risk of long‐term complications associated with different treatment modalities, with the intention of providing valuable insights for clinicians, health policymakers, and medical practitioners to improve patient treatment outcomes and the efficiency of the healthcare system.

By systematically reviewing the relevant literature, this review will offer a comprehensive perspective on the choice of treatment methods for femoral fractures, considering factors such as resource availability, cost‐effectiveness, patient health status, physician experience, patient preferences, and expected clinical outcomes. Moreover, this study will pay special attention to the elderly patient population, whose treatment decision‐making process is often complicated by the presence of other health issues.

## 2 Method

This systematic literature review was conducted in accordance with the PRISMA statement [22]. We selected all types of original studies published in the last 10 years since January 1, 2015. The subjects of the study were patients with femoral fractures who received traction therapy or surgical treatment in resource‐limited areas. The reports included the number of patients, study design type, average age, fracture type, region, treatment method, factors affecting the treatment method, cost‐effectiveness, complications, and contraindications for surgery.

## 3 Inclusion and Exclusion Criteria

### 3.1 Inclusion Criteria

**Study Design**: Randomized controlled trials, cohort studies, case–control studies, cross‐sectional studies, retrospective studies, prospective economic analyses, and other original research.

### 3.2 Study Subjects

**Age**: Adult patients, especially the elderly.

**Diagnosis**: Femoral fractures, including femoral shaft fractures, femoral neck fractures, intertrochanteric fractures, etc.

**Treatment Status**: Patients are receiving bone traction or surgical treatment.

**Region**: Particularly studies conducted in underdeveloped areas.

**Patient Condition**: Elderly patients with severe cardiovascular and cerebrovascular diseases, at risk of ischemic necrosis, or other surgical contraindications.

**Intervention Measures**: Bone traction therapy or surgical treatment.

**Outcome Indicators**: Treatment outcomes, such as fracture healing rates, incidence of complications, mortality, functional recovery of patients, cost‐effectiveness, and complications.

### 3.3 Exclusion Criteria

**Study Type**: Non‐original research such as reviews, editorials, case reports, letters, systematic reviews, guidelines, and protocols.

### 3.4 Study Subjects

Non‐adult patients.

Patients without femoral fractures.

Patients receiving treatments other than bone traction or surgical treatment.

**Intervention Measures**: Studies that do not include bone traction therapy or surgical treatment.

**Data Incompleteness**: Research lacking necessary data or results, which cannot be effectively analyzed.

### 3.5 Search Strategy

A systematic search was conducted in the PubMed, Web of Science, and Cochrane Library databases on November 21, 2023, and updates were performed on May 15, 2024, and September 22, 2024.

**FIGURE 1:**
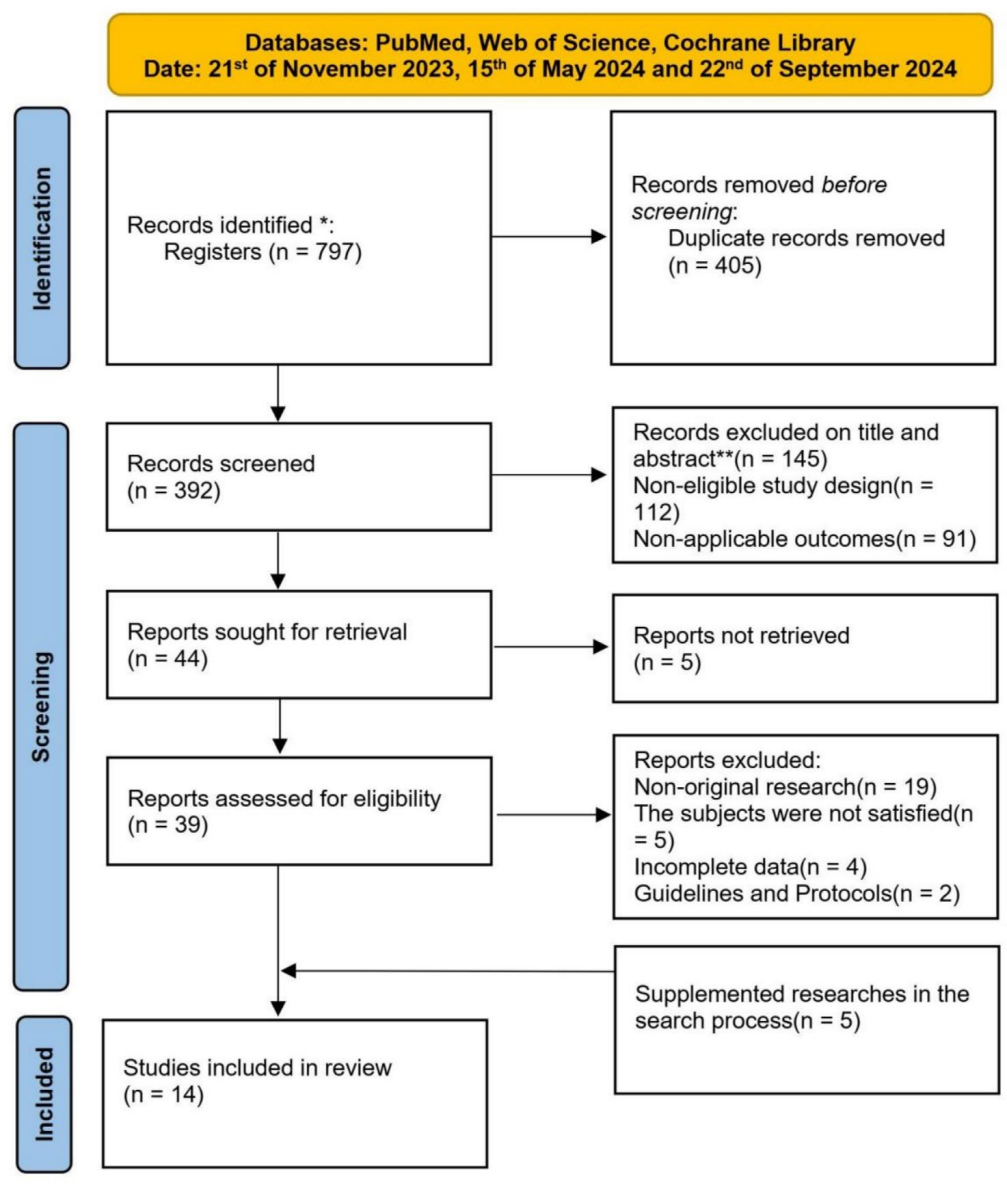
PRISMA 2020 systematic review flow diagram. * Detailed information on the search strategy is reported in the Supporting Information. ** Some studies were excluded for multiple reasons.

With the assistance of an experienced medical librarian, we developed a search strategy that included terms such as “femoral fracture OR traction OR surgery”, “femoral fracture OR traction OR surgery OR efficacy”, “femoral fracture OR traction OR surgery OR cost”, “femoral fracture OR traction OR surgery OR complications”, “femoral fracture OR traction OR surgery OR age”, “femoral fracture OR traction OR surgery OR health”, “femoral fracture OR traction OR surgery OR medical resources”, “femoral fracture OR traction OR surgery OR economics”, “femoral fracture OR traction OR surgery OR personal preference”, etc. The details of the search strategy are shown in Figure 1, and the specific search strategy and results are provided in Supporting Information S1. After duplicates were removed, two reviewers (J.L. and W.W) independently screened the titles and abstracts of the identified records using predefined inclusion and exclusion criteria. Excluded articles were categorized by the reason for exclusion. Disagreements were discussed with a third author (Y.L.). Next, the full texts of the selected articles were evaluated using the same criteria to determine their eligibility for inclusion. The reference lists of included articles were manually searched to identify relevant publications that were missed in the search (Table 1).

**TABLE 1:** Extraction of study characteristics.

Authors	Study design type	Number of patients	Average age	Type of fracture	Region	Treatment method	Influencing factors	Cost‐Effectiveness	Complications	Contraindications for surgery
Fang Pei 2020 [23]	Retrospective cohort study	250	56.4 ± 6.8	142 cases of DP and 108 cases of NDP	Xuzhou, China	All patients underwent CCS fixation. Among them, 80 patients received CR, and 170 patients received OR	Not given	Not given	The incidence rate of ONFH is 16%	Not given
Linda Chokotho 2020 [24]	A prospective multicenter observational study	248 patients were enrolled in the study, of which 187 completed the 1‐year follow‐up. Among them, 55 patients received IMN, and 132 patients received ST	IMN group: 38 years (SD 13) ST group: 40 years (SD 16)	Most fractures were of the AO/OTA type 32A. The proportion of type 32B fractures was higher in the IMN group than in the ST group	Malawi	IMN: All patients were treated with SIGN intramedullary nails. ST: The patients underwent straight leg traction using Steinmann pins	The decision is made by orthopedic clinicians or surgeons, primarily based on the hospital's surgical capabilities	ST treatment for FHF may have a higher OC, including LOS and the impact on FE	IMN: One case experienced non‐union and was treated with nail exchange. ST: Forty patients (30%) converted to IMN treatment during the study due to DU or non‐union	Not given
Brian C. lau 2018 [25]	Prospective, multicenter, observational study	185 patients: 65 in OR group, 120 in ST group	Not given	All patients are CFR (OTA/AO 32)	Malawi	OR group: Intramedullary nail fixation (IMN) ST group: Skeletal traction	Limited surgical resources in Malawi mean many patients wait weeks for surgery. Treatment is decided by the attending doctor or orthopedic clinic official	Using LCM for cost–benefit analysis, including DC and OC. DC includes MC and SF. OC includes TF, PL, UCC, and AC	Not given	Not given
Linda Chokotho 2021 [26]	Economic evaluation study	187 adult patients, with 55 receiving IMN and 132 undergoing ST treatment	Not given	CFR	Malawi	Intramedullary Nail (IMN): Utilizing an intramedullary nail for antegrade insertion without the need for a fracture table or intraoperative fluoroscopy. Skeletal Traction (ST): Performing straight leg extended skeletal traction using a Steinmann pin	Decision by attending doctor or orthopedic clinical officer	IMN treatment cost is lower than ST IMN has a higher QALY than ST	IMN: Main complication is reoperation, occurring in about 1.8% of cases. ST: Primary complication is delayed healing or non‐healing, with 30% needing transition to IMN	Not given
Jefferey Jaya Raj 2021 [27]	Multicenter Retrospective Cohort Study	46	51.5	Garden Classification: 100% are Garden Type III or IV. Pauwels Classification: 67.4% are Pauwels Type III	Terengganu, Malaysia	HRSA: 69.6% THR: 30.4%	Age, type of fracture, fracture reduction status, bone quality, medical history	Not given	AVN: 32.6%, non‐union: 10.9%, Infection: 4.3%, other complications: less than 5%	Conservative treatment, iFNF, iFSF
Nils P Hailer 2016 [28]	A nationwide matched cohort study	24,699 FNF patients undergoing THA 118,518 age‐, sex‐, and residence‐matched controls	Most patients are between the ages of 70–79 (42%)	FNF	Sweden	THA control group non‐THA treated	Not given	Not given	MI CHF PAD CVD HI	Not given
Mohamed Mustafa Diab 2019 [29]	Prospective Observational Economic Analysis Study	The study enrolled 65 patients: 38 received IMN, 27 had ST	IMN group average age: 38 years; ST group average age: 41 years	ICFGF	Malawi	IMN, ST	ST: Common in low‐ and middle‐income countries due to a lack of experts, implants, equipment, and appropriate operating rooms, with IMN considered costly	IMN: Total cost per patient is $596.97, with an average LOS of 36.35 days. ST: Total cost per patient is $678.02, with an average LOS of 61 days	IMN: In low‐ and middle‐income countries, post‐IMN infection rates range from 0.7% to 5%. ST: Non‐union rates for ST are 6%–10%, with infection rates of 11%–43%	S‐OF, DF, LTI
Mohit Bhandari MD 2016 [30]	Two separate, identically designed, randomized, double‐blind, placebo‐controlled phase 3 clinical trials	The study included 159 participants, with 78 receiving teriparatide and 81 on placebo.	Age 70 (range: 50–94 years)	The majority are FNF, with 73% being NDP and 27% being DP	Patients primarily from Asia and Europe, as well as from the United States	All patients received internal fixation with IMN (sliding hip screw or multiple cancellous screws)	Patient age, type of fracture, life expectancy, comorbidities, etc.	Not given	Not given	Other diseases affecting bone metabolism (aside from OP), CA (excluding already treated SC or CC), BsCa
Eileen Tay 2016 [31]	Retrospective study	340	80 years (range 60–100)	Low‐energy FNF and ITF	Singapore	OR (66.5%): ITF: Sliding hip screw or IMN device Non‐displaced FNF: Cancellous bone screw fixation Displaced FNF: Hemiarthroplasty Non‐surgical Treatment (33.5%): Bed‐chair transfer, mechanical DVT prevention	Overall patient health status, ASA score, surgical risk, patient preference	Pneumonia, UTI, PE, Wound Infection.	Not given	Not given
Devin Conway 2019 [32]	Cross‐sectional study	48–1000	Not given	FHF	Tanzania	OR and ST	Hospital size: Smaller hospitals may use traction or referrals; larger hospitals may perform surgery. Patient finances: Surgery can be expensive, may be unaffordable for some. Surgical contraindications	In a resource‐limited setting, using an intermedullary nail (IMN) for femoral shaft fractures is a cost‐effective treatment method	Not given	Not given
Erik J. Kramer 2016 [33]	Prospective observational economic analysis	46	32.4 years (range 18–68 years)	COF: 45 cases (98%) OF: 1 case (2%)	Tanzania	IMN: 42 cases (91%) with SIGN nails, 4 cases (9%) with DePuy Synthes UFN. Approaches: 22 antegrade, 24 retrograde. Anesthesia: 26 general, 20 spinal nerve block	The patient's primary doctor based on their judgment	Average total cost per patient: $530.87 (SD $129.99). Average variable cost: $419.87 (SD $129.99), dominated by ward staff (27.2%), IMN and screws (25.3%), and medical staff (20.1%). Fixed costs: $111.00, covering surgical instruments and support staff.	Not given	Not given
L. Bommireddy2020 [34]	Single‐center retrospective study	53 patients aged 60 and above with FHF	78.7 years (60–96 years range)	81.1% with AO Type A (simple fractures) 9.4% with AO Type B (wedge fractures) 9.4% with AO Type C (complex fractures)	Derbyshire, UK	96.2% had surgery; 3.8% had non‐surgical treatment. IMN was the most common surgery (84.9%), followed by plate fixation (11.3%)	Fracture type and stability, overall patient health and comorbidities, and patient PREFERENCES	Not given	Medical complications occurred in 41.5% of patients, primarily respiratory infections and electrolyte imbalances. Orthopedic complications were seen in 9.4%, mainly non‐union. The 30‐day readmission rate was 19.1%	Not given
Monica Berggren 2016 [35]	Retrospective cohort study	199	82.2 ± 6.2	FNF	Umeå University Hospital, Sweden	Internal fixation (35%) Unilateral hip replacement (56%) Dynamic hip screw (9%) Other (0.5%)	Not given	Not given	Inpatient: Common complications are pneumonia/chest infection. Post‐discharge: Common complications include pneumonia/chest infection, UTIs, and other infections	Rheumatoid arthritis, severe hip osteoarthritis, pathological fracture, severe renal failure
Diamond T. E 2022 [36]	Retrospective study	Not given	29.2 ± 13.8	Closed fractures accounted for 90.1%, while open fractures constituted 9.9%. The prevalent type of open fracture was Type IIIb, making up 57.1%	Nigeria	Open reduction with locked intramedullary nail (62%, *n* = 44/71) Hip spica cast (11.3%, *n* = 8/71) Bone traction (1.4%, *n* = 1/71) External fixator (5.6%, *n* = 4/71) Plate and screw (11.3%, *n* = 8/71) Above‐knee amputation (2.8%, *n* = 2/71)	Fracture type, patient age, available resources and skills, economic status	Intramedullary nailing is costly but reduces hospital stay and complications, aiding early recovery. Hip spica casting is cheaper but can cause joint stiffness and malformation	Incisional infection (5.6%, *n* = 4/71), Nonunion (2.8%, *n* = 2/71), Malunion (1.4%, *n* = 1/71), Osteomyelitis (1.4%, *n* = 1/71), Joint stiffness (1.4%, *n* = 1/71)	Local infection, severe soft tissue injury, bone tumor, severe osteoporosis

Abbreviations: AC, addressing costs; AVN, avascular necrosis of the femoral head; BsCa, baseline serum calcium; CC, cervical cancer; CCS fixation, cannulated compression screw internal fixation; CFR, closed femoral shaft fracture; CHF, congestive heart failure; COF, closed fracture; CR, closed reduction; CVD, cardiovascular disease; DC, direct costs; DF, deep fascial space infection; DP, displaced fracture; DU, delayed union; FE, family economy; FFS, femoral shaft fracture; FNF, femoral neck fracture; HI, hypertensive intracerebral hemorrhage; HRSA, hip resurfacing surgery arthroplasty; ICFGF, isolated closed femoral shaft fracture; iFNF, iatrogenic femoral neck fracture; iFSF, ipsilateral femoral shaft fracture; IMN, intramedullary nail fixation; ITF, inter‐trochanteric fracture; LCM, life cycle management context; LOS, length of stay; LTI, lower thoracic infection; MC, medical costs; MI, myocardial infarction; NDP, non‐displaced fracture; OC, indirect costs; OF, severe open fracture; ONFH, osteonecrosis of the femoral head; OP, osteoporosis; OR, open reduction; PAD, peripheral arterial disease; PE, pulmonary embolism; PL, productivity loss; QALY, quality‐adjusted life year; SC, skin cancer; SF, surgical fees; S‐OF, soft tissue infection; ST, skeletal traction; TF, transportation fees; THR, total hip replacement; UCC, uncompensated care costs; UTI, urinary tract infection.

### 3.6 Data Extraction and Study Characteristics

Data extraction was performed by two independent reviewers (J.L. and W.W.) using a previously designed data extraction form. Any discrepancies were discussed with a third author (Y.L.). The following data were extracted from the included studies: number of patients, mean age, type of fracture, region, treatment modalities, factors affecting treatment modalities, cost‐effectiveness, complications, and surgical contraindications. Among them, cost‐effectiveness, complications, and surgical contraindications are the key factors we focus on, while the number of patients, mean age, type of fracture, region, treatment modalities, and factors affecting treatment modalities are also of concern to us. Cost‐effectiveness, complications, and surgical contraindications play a crucial role in the selection and trade‐off between surgery and traction, and factors affecting treatment modalities also influence the choice of treatment methods.

### 3.7 Risk of Bias in Studies

**FIGURE 2:**
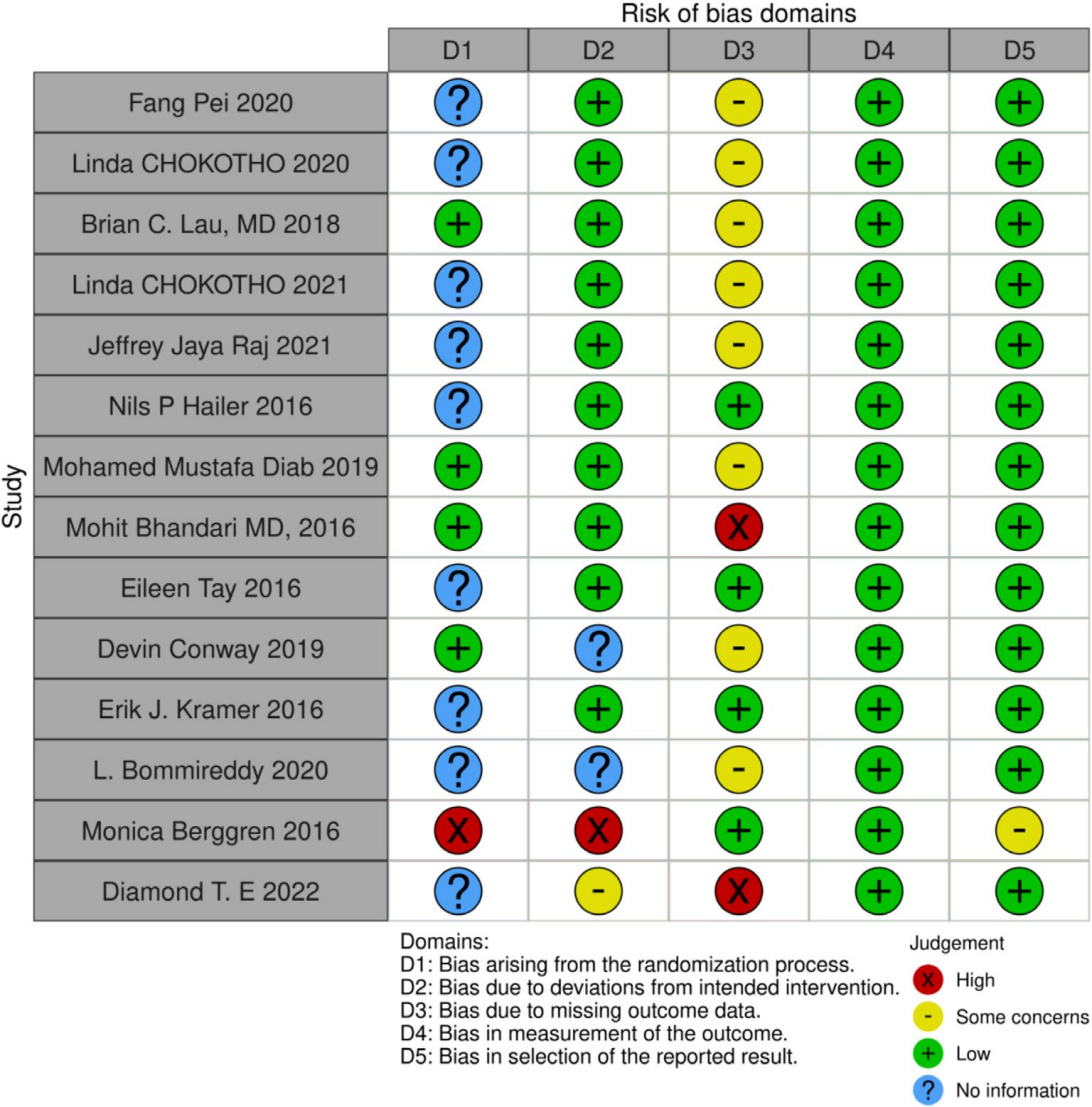
ROB2 assessment results graph.

The Grading of Recommendations Assessment, Development, and Evaluation (GRADE) approach (Table 2) was used to determine the certainty of the summary estimates of the association between intervention factors and future outcomes, including five domains: risk of bias, imprecision, inconsistency of study results, indirectness, and publication bias [37]. The Risk of Bias in Intervention Studies (ROB2) tool (Figure 2) was used to assess the risk of bias in individual studies [38]. Inconsistency of study results across research was assessed by analyzing the forest plots and the statistical heterogeneity measure (I^2^) among the studies included in the meta‐analysis. The imprecision of the estimates was based on whether the 95% confidence intervals (CI) excluded a ratio of 1 and the width of the CI. Indirectness was assessed by comparing the study populations and outcomes across studies. The possibility of publication bias was visually assessed through a funnel plot (Supporting Information S2), and sensitivity analyses were conducted for each influencing factor (Supporting Information S3).

**TABLE 2:** Evidence quality using the GRADE method^a^.

Predictive factor	Risk of bias	Inconsistency	Imprecision	Indirectness	Publication bias	Estimated certainty
Number of patients	Low	Low	Moderate	Low	Low	Low
Average age (> 65, < 65)	Low	Low	Moderate	Low	Low	Low
Health status	Moderate	Moderate	Moderate	Moderate	Moderate	Moderate
Type of fracture	Moderate	Moderate	Moderate	Moderate	Moderate	Moderate
Region (underdeveloped, developed)	High	High	Low	High	High	High
Physician's choice	Low	Low	Moderate	Low	Low	Low
Surgical capability	Moderate	Moderate	Moderate	Moderate	Moderate	Moderate
Equipment condition	Low	Low	Moderate	Low	Low	Low
Medical resources	High	High	Low	High	High	High

^a^ The estimated certainty is graded according to the method previously described by Foroutan et al. [37]: High: We are very confident that the variation in risk associated with the prognostic factor (probability of future events with/without the prognostic factor) is close to the estimated value. Moderate: We have moderate confidence that the variation in risk associated with the prognostic factor (probability of future events with/without the prognostic factor) is likely to be close to the estimated value, but there is a possibility of substantial differences. Low: We have limited certainty in the estimated value: the variation in risk associated with the prognostic factor (probability of future events with/without the prognostic factor) may have substantial differences from the estimated value. Very low: We have very little certainty in the estimated value: the variation in risk associated with the prognostic factor (probability of future events with/without the prognostic factor) is likely to have substantial differences from the estimated value. Not assessed: Not assessed because it was evaluated in only one study.

### 3.8 Data Synthesis

RevMan 5 software [39] was used to summarize the univariate odds ratios (ORs) for predictors using a random‐effects model. The I^2^ statistic was used to measure the heterogeneity of study results, representing the percentage of variation between studies due to statistical heterogeneity, with the following criteria: 0% to 40% (probably unimportant); 30% to 60% (may represent moderate heterogeneity); 50% to 90% (may represent significant heterogeneity); 75% to 100% (considerable heterogeneity) [40].

## 4 Results

### 4.1 Study Selection and Characteristics

The preliminary search of the databases yielded a total of 797 records (Figure 1). After removing duplicates, 392 potentially eligible study articles were identified. Records excluded based on titles and abstracts (*n* = 145), ineligible study design (*n* = 112), and inapplicable outcomes (*n* = 91) resulted in 44 articles being retrieved for full‐text review. After excluding 5 articles that could not be retrieved, 39 reports were eligible for qualification assessment. Non‐original studies (*n* = 19), studies with ineligible subjects (*n* = 5), studies with incomplete data (*n* = 4), and guidelines and protocols (*n* = 2) were excluded. An additional 5 articles were identified through references during the retrieval process. Finally, 14 articles met the inclusion criteria and were included in this systematic review.

All included studies were original research, comprising randomized controlled trials (RCTs), cohort studies, cross‐sectional studies, retrospective studies, and prospective economic analyses. The overall sample size of the included papers ranged from 46 to 24,699 patients. The average age range was from 29.2 to 80 years. Five studies [27, 28, 30, 33, 35] included only surgical treatment, while the other 9 included both surgical treatment and bone traction treatment [24, 25, 26, 29, 31, 32, 33, 34, 36]. Most studies were conducted in less developed countries, primarily including Malawi [24, 25, 26, 29], Tanzania [32, 33], Nigeria [36], Terengganu, Malaysia [27], and Xuzhou, China [23], with a few studies in more developed regions, including Sweden [28, 35], Singapore [31], the United Kingdom [34], and the United States [30]. The main types of fractures included femoral shaft fractures, femoral neck fractures, intertrochanteric fractures, etc. Most studies analyzed cost‐effectiveness, complications, and contraindications for surgery.

### 4.2 Bias Risk and Quality of Evidence Assessment

The ROB2 (Risk of Bias 2) tool is used to assess the risk of bias in randomized controlled trials to determine the reliability and applicability of study results. It consists of five domains: D1 for bias due to the randomization process, D2 for bias due to deviation from intended interventions, D3 for bias due to missing outcome data, D4 for bias in the measurement of the outcome, and D5 for bias in the selection of the reported result. Each domain is assessed with four judgments: High indicates a high risk of bias that may seriously affect the reliability of the results; Some concerns suggest a certain level of risk of bias that may impact the reliability of the results; Low signifies a low risk of bias with higher result reliability; and No information means there is insufficient information to assess the risk of bias in the study.

### 4.3 Factors Influencing Treatment Modality Selection

**FIGURE 3:**
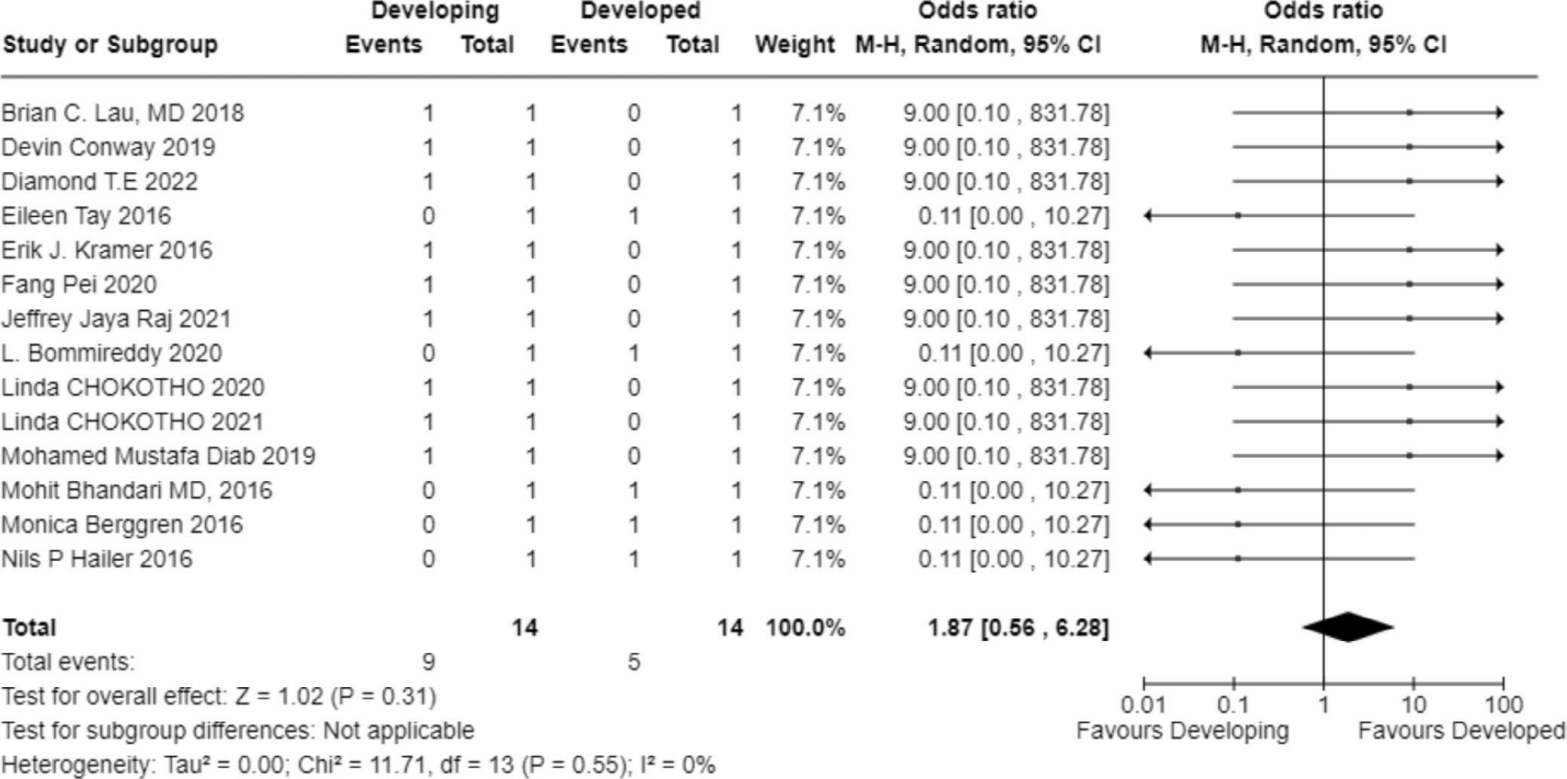
Forest plot of regional resource availability.

**FIGURE 4:**
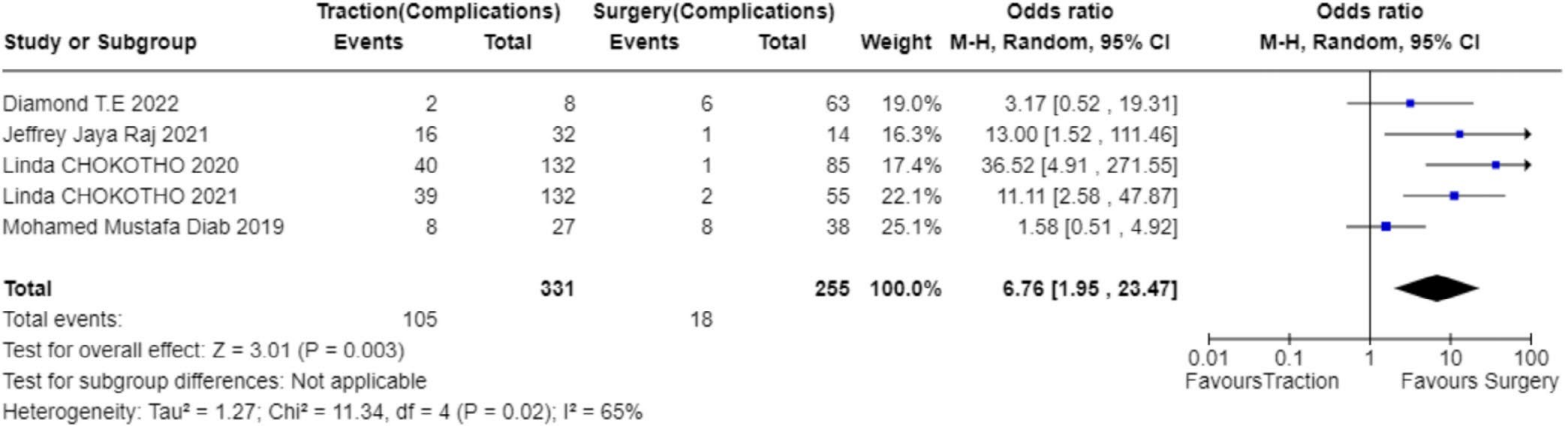
Forest plot of complications.

**FIGURE 5:**
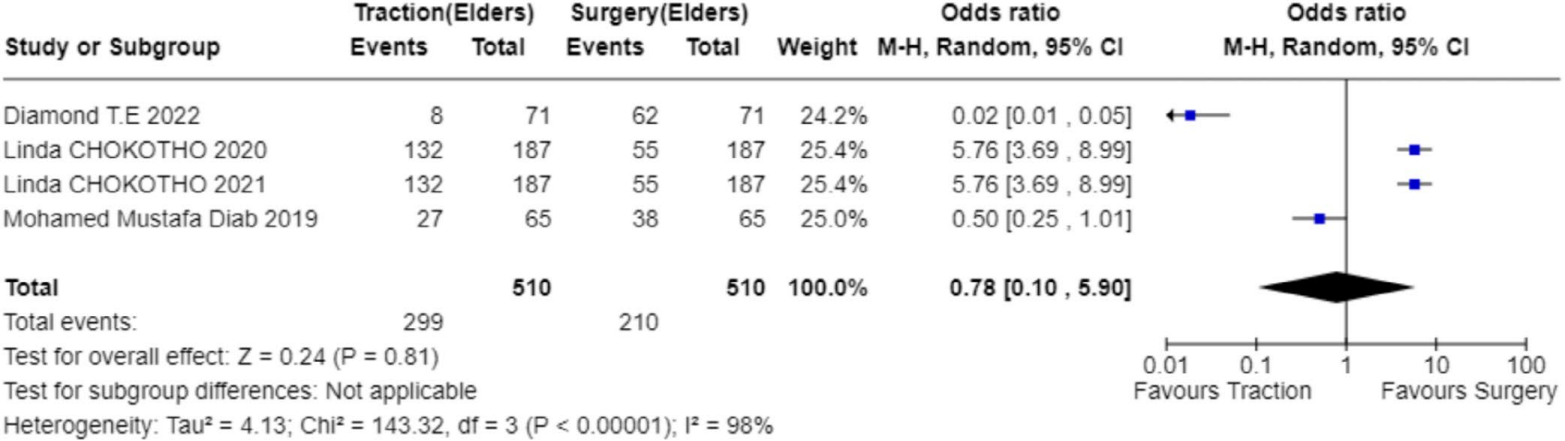
Forest plot for selection of elderly patients.

In exploring the factors that influence the choice of treatment modality, our analysis included studies from multiple regions, covering environments with different economic levels and medical resources. The results indicate that the availability of resources is the primary factor determining the treatment modality (OR 1.87; 95% CI 0.56–6.28; Figure 3) [23] (OR 9.00; 95% CI 0.10–831.78; Figure 3) [24] (OR 9.00; 95% CI 0.10–831.78; Figure 3) [25] (OR 9.00; 95% CI 0.10–831.78; Figure 3) [26] (OR 9.00; 95% CI 0.10–831.78; Figure 3) [27] (OR 9.00; 95% CI 0.10–831.78; Figure 3) [28] (OR 0.11; 95% CI 0.00–10.27; Figure 3) [29] (OR 9.00; 95% CI 0.10–831.78; Figure 3) [30] (OR 0.11; 95% CI 0.00–10.27; Figure 3) [31] (OR 0.11; 95% CI 0.00–10.27; Figure 3) [32] (OR 9.00; 95% CI 0.10–831.78; Figure 3) [33] (OR 9.00; 95% CI 0.10–831.78; Figure 3) [34] (OR 9.00; 95% CI 0.10–831.78; Figure 3) [35] (OR 0.11; 95% CI 0.00–10.27; Figure 3) [36] (OR 9.00; 95% CI 0.10–831.78; Figure 3). For instance, in a study focused on a hospital in Malawi, due to limited surgical resources, only a minority of patients could receive surgery within 6 weeks, resulting in the majority receiving bone traction treatment [25] (OR 9.00; 95% CI 0.10–831.78; Figure 3). Additionally, clinical outcomes considerations, such as fracture healing rates and infection rates, significantly influenced the choice of treatment modality in five studies (OR 6.76; 95% CI 1.95–23.47; Figure 4) [24] (OR 36.52; 95% CI 4.91–271.55; Figure 4) [26] (OR 11.11; 95% CI 2.58–47.87; Figure 4) [27] (OR 13.00; 95% CI 1.52–111.46; Figure 4) [29] (OR 1.58; 95% CI 0.51–4.92; Figure 4) [36] (OR 3.17; 95% CI 0.52–19.31; Figure 4). Elderly patients tended to choose non‐surgical treatment to reduce surgical risks, which was reflected in four studies (OR 0.78; 95% CI 0.10–5.90; Figure 5) [24] (OR 5.76; 95% CI 3.69–8.99; Figure 5) [26] (OR 5.76; 95% CI 3.69–8.99; Figure 5) [29] (OR 0.50; 95% CI 0.25–1.01; Figure 5) [36] (OR 0.02; 95% CI 0.01–0.05; Figure 5). Economic factors also played a role, with one study indicating that patients with poorer economic status were more likely to choose non‐surgical treatment [32] (OR 37.73; 95% CI 29.31–48.58; Figure 9).

### 4.4 Treatment Options in Underdeveloped Areas

Studies on treatment options in underdeveloped areas highlight the impact of resource constraints on clinical decision‐making. A prospective economic analysis conducted in Malawi showed that, despite intramedullary nailing (IMN) being superior in clinical outcomes to bone traction, the higher cost of IMN makes bone traction the more common choice [29]. Additionally, a study across different hospitals found that hospital size and resources significantly influenced treatment options, with smaller hospitals more likely to use bone traction or refer patients, while larger hospitals were more likely to perform surgical fixation [32].

### 4.5 Selection for Elderly Patients With Surgical Contraindications

For elderly patients with surgical contraindications, the study results suggest that elderly patients may tend to non‐surgical treatment to reduce surgical risk (OR 0.78; 95% CI 0.10–5.90; Figure 5) [24] (OR 5.76; 95% CI 3.69–8.99; Figure 5) [26] (OR 5.76; 95% CI 3.69–8.99; Figure 5) [29] (OR 0.50; 95% CI 0.25–1.01; Figure 5) [36] (OR 0.02; 95% CI 0.01–0.05; Figure 5). However, non‐surgical treatment may not always be the best choice. A study in Singapore found that elderly patients in the non‐surgical treatment group had a mortality risk 4.49 times and 3.33 times higher than the surgical treatment group at 1 and 2 years of follow‐up, respectively [31]. In a nationwide study in Sweden, although elderly patients undergoing surgery had a higher mortality rate in the early postoperative period, patients under the age of 80 with fewer comorbidities had a relatively lower risk of early death [28]. This indicates that when selecting a treatment plan, it is necessary to consider the patient's overall health status and surgical risk.

## 5 Discussion

In this systematic review, we delve into the selection of treatment strategies for adult patients and elderly patients with surgical contraindications suffering from femoral fractures, especially comparing the pros and cons of surgery versus traction therapy in resource‐limited settings.

### 5.1 Factors Influencing Treatment Modality Selection

**FIGURE 6:**
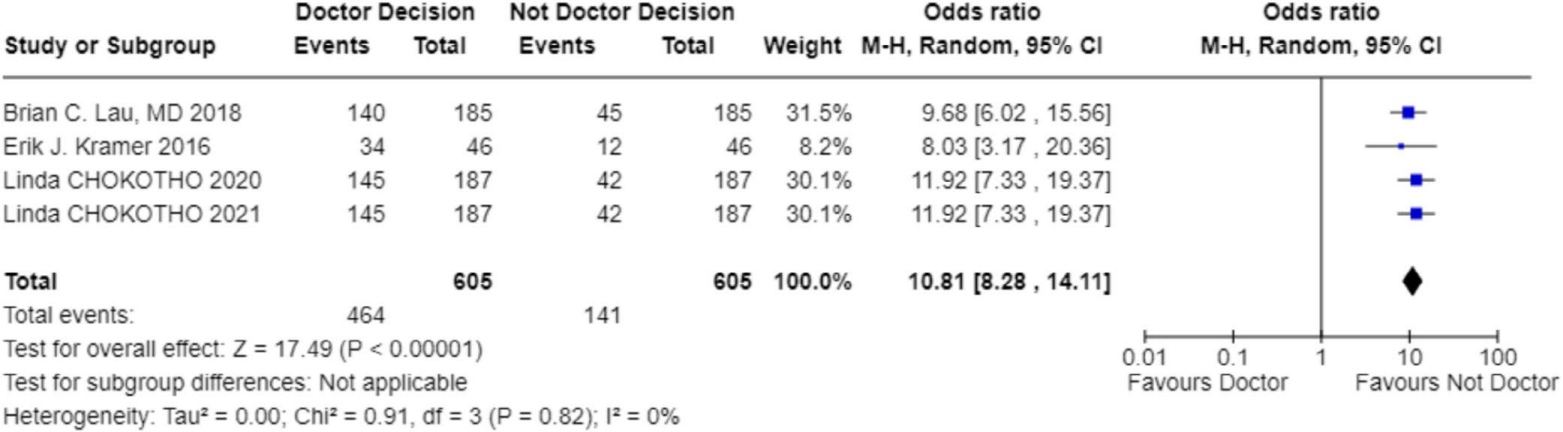
Forest plot for physician decision‐making.

**FIGURE 7:**
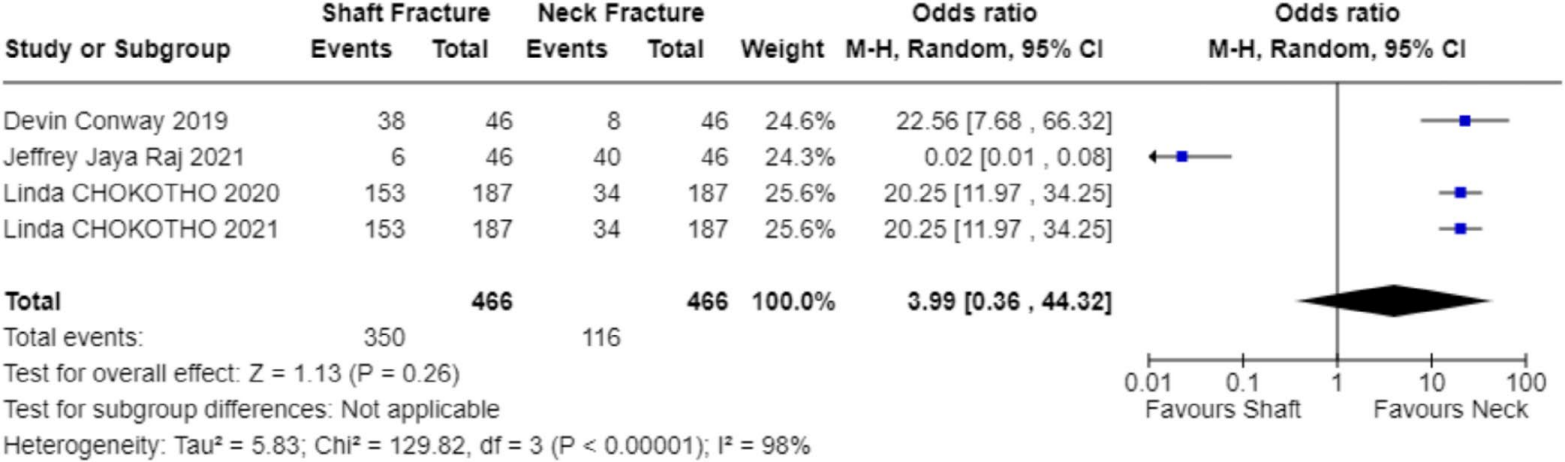
Forest plot of fracture types.

**FIGURE 8:**
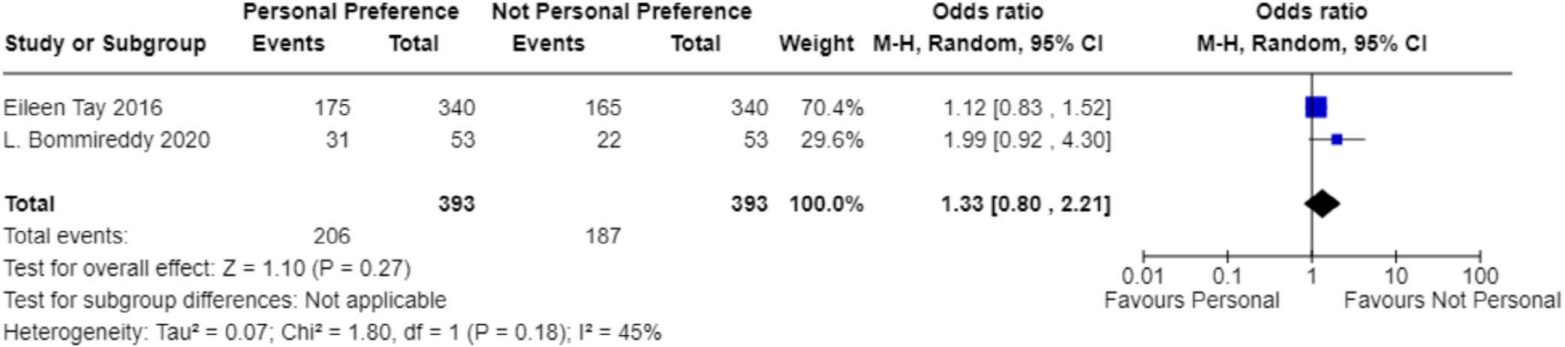
Forest plot of individual preferences.

**FIGURE 9:**
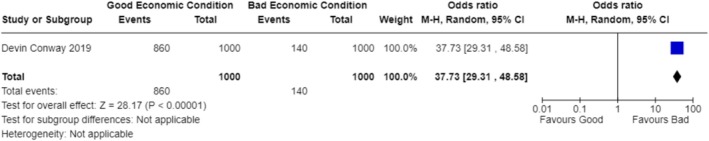
Forest plot of economic status.

When analyzing the factors influencing treatment strategy selection, multiple studies have identified key decision‐making points. The availability of resources plays a particularly crucial role in treatment decisions in low‐ and middle‐income countries (OR 1.87; 95% CI 0.56–6.28; Figure 3) [23] (OR 9.00; 95% CI 0.10–831.78; Figure 3) [24] (OR 9.00; 95% CI 0.10–831.78; Figure 3) [25] (OR 9.00; 95% CI 0.10–831.78; Figure 3) [26] (OR 9.00; 95% CI 0.10–831.78; Figure 3) [27] (OR 9.00; 95% CI 0.10–831.78; Figure 3) [28] (OR 0.11; 95% CI 0.00–10.27; Figure 3) [29] (OR 9.00; 95% CI 0.10–831.78; Figure 3) [30] (OR 0.11; 95% CI 0.00–10.27; Figure 3) [31] (OR 0.11; 95% CI 0.00–10.27; Figure 3) [32] (OR 9.00; 95% CI 0.10–831.78; Figure 3) [33] (OR 9.00; 95% CI 0.10–831.78; Figure 3) [34] (OR 9.00; 95% CI 0.10–831.78; Figure 3) [35] (OR 0.11; 95% CI 0.00–10.27; Figure 3) [36] (OR 9.00; 95% CI 0.10–831.78; Figure 3). Specifically, four studies pointed out that in underdeveloped areas, the mode of treatment is often decided by orthopedic surgeons or doctors based on the actual situation (OR 10.81; 95% CI 8.28–14.11; Figure 6) [24], (OR 11.92; 95% CI 7.33–19.37; Figure 6) [25], (OR 9.68; 95% CI 6.02–15.56; Figure 6) [26], (OR 11.92; 95% CI 7.33–19.37; Figure 6) [33], (OR 8.03; 95% CI 3.17–20.36; Figure 6). Additionally, one study found that the choice of treatment is closely related to the level of expertise of the specialists [29]. For instance, in Malawi, due to a shortage of surgical resources, patients often have to wait for weeks to undergo surgery, leading to bone traction becoming a more common treatment choice (OR 9.00; 95% CI 0.10–831.78; Figure 3) [25]. The clinical outcomes and risks of complications associated with different treatment modalities, including fracture healing rates, infection rates, non‐union rates, and rates of reoperation, are also significant factors considered by doctors and patients (OR 6.76; 95% CI 1.95–23.47; Figure 4) [24] (OR 36.52; 95% CI 4.91–271.55; Figure 4) [26] (OR 11.11; 95% CI 2.58–47.87; Figure 4) [27] (OR 13.00; 95% CI 1.52–111.46; Figure 4) [29] (OR 1.58; 95% CI 0.51–4.92; Figure 4) [36] (OR 3.17; 95% CI 0.52–19.31; Figure 4). The age of the patient and overall health status also influence the choice of treatment, as elderly patients may be more inclined towards non‐surgical treatment to reduce surgical risks (OR 0.78; 95% CI 0.10–5.90; Figure 5) [24] (OR 5.76; 95% CI 3.69–8.99; Figure 5) [26] (OR 5.76; 95% CI 3.69–8.99; Figure 5) [29] (OR 0.50; 95% CI 0.25–1.01; Figure 5) [36] (OR 0.02; 95% CI 0.01–0.05; Figure 5). Five studies showed that the patient's age, health status, and type of fracture are key factors in determining treatment choices (OR 3.99; 95% CI 0.36–44.32; Figure 7) [24], (OR 20.25; 95% CI 11.97–34.25; Figure 7) [26], (OR 20.25; 95% CI 11.97–34.25; Figure 7) [27], (OR 0.02; 95% CI 0.01–0.08; Figure 7) [32], (OR 22.56; 95% CI 7.68–66.32; Figure 7). Two studies indicated that the personal preferences of the patients also play a role in treatment decisions (OR 1.33; 95% CI 0.80–2.21; Figure 8) [31] (OR 1.12; 95% CI 0.83–1.52; Figure 8) [34] (OR 1.99; 95% CI 0.92–4.30; Figure 8). Another study showed that the patient's economic status is also an important factor affecting the choice of treatment [32] (OR 37.73; 95% CI 29.31–48.58; Figure 9). These findings suggest that the choice of treatment is a complex decision‐making process involving multiple factors.

Gender differences and different fracture types may also be an impacting factor in the trade‐offs between surgery and traction. Ceolin et al. [41] found that men may experience slower functional recovery after hip fracture, with higher rates of readmission and mortality compared to women, while women may require more family and social support after hip fracture. Gender differences may have some impact on functional recovery and prognosis in patients with hip fractures, potentially necessitating different treatment approaches based on gender. However, further investigation is needed to develop more effective treatment strategies tailored to different genders. In the study by Biz [42] and others, Type I and Type II fractures (undisplaced and partially displaced fractures) are typically managed conservatively, which includes rest, avoidance of weight‐bearing, the use of crutches, etc., supplemented by medication to improve bone metabolism. Type II fractures (completely displaced fractures) require surgical treatment, usually with the use of DHS (Dynamic Hip Screw) plates for internal fixation to stabilize the fracture and promote healing. The greater the degree of displacement in the fracture, the more difficult and risky the surgical treatment becomes, and the higher the risk of postoperative avascular necrosis (AVN) of the femoral head. Early diagnosis and treatment are key to preventing AVN, and it is recommended that surgical treatment be performed within 6 h of the onset of symptoms. This illustrates that the different classifications of fractures will also affect the choice of treatment methods.

In the previous section, we noted that both surgical and non‐surgical treatments are associated with complications. In this part, we delve into the impact of these complications on the quality of life for patients. In surgical treatment, common complications include bleeding, infection, thrombosis, and postoperative dysfunction. These complications can lead to extended hospital stays, a more complicated recovery process, and may even necessitate additional surgery to address these issues. On the other hand, non‐surgical treatments also have their specific complications, which mainly include lower limb venous thrombosis, pneumonia, and bedsores. Due to the prolonged periods of bed rest required, patients may experience muscle atrophy and joint stiffness, thereby affecting their ability to perform daily activities. Moreover, patients undergoing non‐surgical treatment often face higher medical and economic burdens during the treatment process, which can lead to increased psychological stress and further impact their quality of life.

In comparing the existing literature with our study's findings, we found that resource availability is a key factor influencing treatment choice, which aligns with the findings of Diab et al. [29] in their 2019 study in Malawi. This study highlighted the phenomenon where limited surgical resources led to skeletal traction becoming the treatment of choice. However, our data also suggest that even in resource‐constrained areas, more cost‐effective treatment methods, such as intramedullary nailing (IMN) [32], can be promoted through effective resource allocation and cost–benefit analysis. Studies by Gosselin et al. [43] in 2009 and Opondo et al. [44] in 2013 also found that despite the higher initial cost of IMN, the overall cost may be lower in the long term due to better clinical outcomes and shorter hospital stays.

Our research results also reveal the importance of patient preference in treatment choice (OR 1.33; 95% CI 0.80–2.21; Figure 8) [31] (OR 1.12; 95% CI 0.83–1.52; Figure 8) [34] (OR 1.99; 95% CI 0.92–4.30; Figure 8), an area not fully explored in the existing literature. This indicates that treatment decisions are increasingly needing to be patient‐centered, considering individualized needs and preferences. Furthermore, our findings are consistent with the studies by Tay [31] in 2016 and Hailer et al. [28] in 2016, which indicated that non‐surgical treatment may carry a higher mortality risk for elderly patients. Our research further suggests that for certain elderly patients, surgical treatment may be a better option through careful assessment of surgical risks and potential long‐term benefits [28, 31].

The impact of regional and healthcare system differences on treatment choices was also highlighted in our study, in line with the findings of Bommireddy et al. [34]. These differences can affect the availability of treatments and the choices made by patients. Inconsistencies may arise from a variety of factors, including study design, sample size, characteristics of the patient population, and differences in healthcare systems across regions.

### 5.2 Treatment Options in Underdeveloped Areas

In underdeveloped areas, the choice of treatment is influenced by a combination of factors, which has been made clear in several studies. The limited availability of resources is one of the main factors affecting treatment choices. For instance, in Malawi, the scarcity of surgical resources makes intramedullary nailing and other surgical options not always feasible, leading to bone traction becoming the primary method of treatment [29]. Moreover, cost‐effectiveness analyses indicate that although intramedullary nailing may have better clinical outcomes in certain cases, it is less cost‐effective than bone traction, a conclusion confirmed by a prospective economic analysis study in Malawi [29]. Clinical outcomes and complications, including fracture healing rates, infection rates, non‐union rates, and rates of reoperation, are also significant factors influencing the choice of treatment by both doctors and patients (OR 6.76; 95% CI 1.95–23.47; Figure 4) [24] (OR 36.52; 95% CI 4.91–271.55; Figure 4) [26] (OR 11.11; 95% CI 2.58–47.87; Figure 4) [27] (OR 13.00; 95% CI 1.52–111.46; Figure 4) [29] (OR 1.58; 95% CI 0.51–4.92; Figure 4) [36] (OR 3.17; 95% CI 0.52–19.31; Figure 4). For example, a study conducted in Malawi found that patients treated with intramedullary nailing had better early quality of life and functional recovery compared to those treated with bone traction [26]. These research findings suggest that in resource‐constrained environments, the most appropriate treatment choice requires a comprehensive consideration of clinical effectiveness and complications (OR 6.76; 95% CI 1.95–23.47; Figure 4) [24] (OR 36.52; 95% CI 4.91–271.55; Figure 4) [26] (OR 11.11; 95% CI 2.58–47.87; Figure 4) [27] (OR 13.00; 95% CI 1.52–111.46; Figure 4) [29] (OR 1.58; 95% CI 0.51–4.92; Figure 4) [36] (OR 3.17; 95% CI 0.52–19.31; Figure 4), patient condition (OR 3.99; 95% CI 0.36–44.32; Figure 7) [24] (OR 20.25; 95% CI 11.97–34.25; Figure 7) [26] (OR 20.25; 95% CI 11.97–34.25; Figure 7) [27] (OR 0.02; 95% CI 0.01–0.08; Figure 7) [32] (OR 22.56; 95% CI 7.68–66.32; Figure 7) and the accessibility of medical resources (OR 1.87; 95% CI 0.56–6.28; Figure 3) [23] (OR 9.00; 95% CI 0.10–831.78; Figure 3) [24] (OR 9.00; 95% CI 0.10–831.78; Figure 3) [25] (OR 9.00; 95% CI 0.10–831.78; Figure 3) [26] (OR 9.00; 95% CI 0.10–831.78; Figure 3) [27] (OR 9.00; 95% CI 0.10–831.78; Figure 3) [28] (OR 0.11; 95% CI 0.00–10.27; Figure 3) [29] (OR 9.00; 95% CI 0.10–831.78; Figure 3) [30] (OR 0.11; 95% CI 0.00–10.27; Figure 3) [31] (OR 0.11; 95% CI 0.00–10.27; Figure 3) [32] (OR 9.00; 95% CI 0.10–831.78; Figure 3) [33] (OR 9.00; 95% CI 0.10–831.78; Figure 3) [34] (OR 9.00; 95% CI 0.10–831.78; Figure 3) [35] (OR 0.11; 95% CI 0.00–10.27; Figure 3) [36] (OR 9.00; 95% CI 0.10–831.78; Figure 3).

### 5.3 Selection for Elderly Patients With Surgical Contraindications

When considering elderly patients with surgical contraindications, the choice of treatment becomes even more complex and requires a comprehensive assessment of the patient's overall health status, type of fracture, and comorbidities (OR 3.99; 95% CI 0.36–44.32; Figure 7) [24] (OR 20.25; 95% CI 11.97–34.25; Figure 7) [26] (OR 20.25; 95% CI 11.97–34.25; Figure 7) [27] (OR 0.02; 95% CI 0.01–0.08; Figure 7) [32] (OR 22.56; 95% CI 7.68–66.32; Figure 7). Some studies suggest that in the elderly population, particularly those with severe comorbidities or other health issues, non‐surgical treatment methods, such as hip arthroplasty or internal fixation surgery, may be more favored due to their lower surgical risks (OR 0.78; 95% CI 0.10–5.90; Figure 5) [24] (OR 5.76; 95% CI 3.69–8.99; Figure 5) [26] (OR 5.76; 95% CI 3.69–8.99; Figure 5) [29] (OR 0.50; 95% CI 0.25–1.01; Figure 5) [36] (OR 0.02; 95% CI 0.01–0.05; Figure 5). For instance, the study by Tay et al. [31] in Singapore found that the mortality risk in the non‐surgical treatment group was significantly higher than in the surgical treatment group among elderly patients, highlighting that surgical intervention may be more appropriate in certain cases even within the elderly population. Additionally, a nationwide study in Sweden by Hailer et al. [28] found that while the early postoperative mortality rate was relatively high in elderly patients, the early mortality risk after total hip arthroplasty (THA) was relatively low for patients under 80 years old with no significant comorbidities, and other treatment strategies may need to be considered for older patients or those with more comorbidities. These studies indicate that when selecting treatment plans for elderly patients with surgical contraindications, doctors must weigh the potential benefits and risks of surgery and consider the long‐term impacts of non‐surgical treatments.

### 5.4 Limitations of Our Study

When interpreting the results of this study, we must acknowledge a range of potential limitations. Firstly, most studies included in this review employed observational designs, which may introduce selection bias and confounding variables, particularly when comparing different treatment modalities such as intramedullary nailing (IMN) versus skeletal traction. Since patients were not randomly assigned to treatment groups, treatment choices may be influenced by multiple unmeasured factors, such as physician preference and specific patient conditions.

Secondly, limitations in data collection are also worth noting, especially in studies that rely on retrospective data. Such studies may face challenges with data completeness and accuracy as they depend on the integrity of medical records, which may not always be complete. Additionally, issues with sample size and study representativeness limit the interpretation of our results. Some studies may have small sample sizes, and the selection of patients may be influenced by specific medical environments, which require caution when generalizing these findings to a broader patient population.

Furthermore, the length of follow‐up is particularly important for assessing treatment outcomes. Some studies may have follow‐up periods that are too short to fully observe long‐term treatment outcomes, such as long‐term mortality and complications in elderly patients after hip surgery. The limitations of economic analyses should also not be overlooked, as many studies may fail to fully consider all relevant costs and benefits, such as the cost of patients' time and improvements in quality of life.

In terms of statistical analysis, some studies may suffer from insufficient statistical power, which prevents them from detecting true differences between treatment modalities. Additionally, the failure to adjust for potential confounders such as age, gender, and comorbidities may have affected the accuracy of the results.

Finally, while certain associations have been observed, such as the link between IMN and improved quality of life, establishing causality remains challenging. Many studies can only demonstrate correlation between variables, not direct causation, suggesting that other unmeasured factors may also be at play.

### 5.5 Strengths of Our Study

The results of this review emphasize the importance of considering a multitude of factors in clinical practice and provide valuable insights for healthcare decision‐makers to improve patient treatment outcomes and the efficiency of healthcare systems. Through in‐depth analysis of the factors influencing treatment modality choices, we found that resource availability, cost‐effectiveness, and clinical outcomes significantly impact treatment decisions. For instance, in resource‐constrained environments, despite higher initial costs, intramedullary nailing (IMN) may be more cost‐effective in the long run due to better clinical outcomes and shorter hospital stays. Additionally, physician expertise, patient preferences, and hospital surgical capacity are also important considerations.

These findings have potential implications for clinicians, health policymakers, and practitioners. They can assist clinicians in making treatment choices that are more aligned with patients' needs and economic status when faced with femoral neck or shaft fractures. Furthermore, by optimizing resource allocation and developing evidence‐based health policies, decision‐makers can enhance the quality and efficiency of healthcare services.

At the same time, these results also suggest a need for more efforts in patient education to ensure that patients can fully understand the potential outcomes of different treatment options, enabling them to make more informed decisions. Lastly, these findings also provide direction for future research, particularly in how patient preferences influence treatment choices and how to further improve surgical safety and reduce costs. Future research should employ more rigorous designs, including randomized controlled trials, to reduce bias and enhance the reliability of the results. Additionally, long‐term follow‐up and comprehensive economic analyses will help to more accurately assess the benefits of different treatment modalities.

## 6 Conclusion

The selection of treatment modalities under varying conditions is a multifactorial and multidimensional decision‐making process that requires a comprehensive consideration of resource availability, cost‐effectiveness, patient health status, physician experience, patient preferences, and anticipated clinical outcomes. In resource‐constrained environments, particularly among elderly patients, this decision‐making process is especially complex and necessitates a careful balancing of the risks and benefits of surgical versus non‐surgical treatments.

## Author Contributions

Jiarui Li and Zhu Guo are co‐first authors. They conceptualized the study, conducted experiments, analyzed data, and drafted the manuscript. Tianrui Wang and Hongfei Xiang assisted in study design, data collection, and manuscript writing. Kunyue Xing contributed to data interpretation and manuscript revision. Wenzhuo Wang and Yaowei Liu helped with data collection and experiments. Jiyao Xing and Jingdong Wang aided in experimental design and data analysis. Bohua Chen and Dongming Xing supervised the project, secured funding, and provided critical manuscript revisions. Xiaolin Wu, the corresponding author, supervised the project, secured funding, and coordinated the collaboration, significantly revising the manuscript. All authors reviewed and approved the final manuscript.

## Conflicts of Interest

The authors declare no conflicts of interest.

## Supporting information


**Data S1.** PRISMA 2020 Checklist.

**Data S2.** PRISMA 2020 flow diagram for new systematic reviews which included searches of databases and registers only.

**Data S3.** Specific Search.
