# Supplementary material for: Treatment Trade‐Offs and Choices for Femoral Fractures: A Systematic Review and Meta‐Analysis
Source: Orthop Surg. 2025 Mar 25;17(5):1298–313. doi: 10.1111/os.70001 (PMC12050177; doi:10.1111/os.70001)
Supplement: Supplementary file 2 — Data S2. PRISMA 2020 flow diagram for new systematic reviews which included searches of databases and registers only. [file OS-17-1298-s003.docx]

**Databases: PubMed, Web of Science, Cochrane Library**

**Date: 21^st^ of November 2023, 15^th^ of May 2024 and 22^nd^ of September 2024**

Records removed *before screening*:

Duplicate records removed (n = 405)

Records identified *:

Registers (n = 797)

**Identification**

Records excluded on title and abstract**(n = 145)

Non-eligible study design(n = 112)

Non-applicable outcomes(n = 91)

Records screened

(n = 392)

Reports sought for retrieval

(n = 44)

Reports not retrieved

(n = 5)

**Screening**

Reports excluded:

Non-original research(n = 19)

The subjects were not satisfied(n = 5)

Incomplete data(n = 4)

Guidelines and Protocols(n = 2)

Reports assessed for eligibility

(n = 39)

Supplemented researches in the search process(n = 5)

Studies included in review

(n = 14)

**Included**

*Consider, if feasible to do so, reporting the number of records identified from each database or register searched (rather than the total number across all databases/registers).

**If automation tools were used, indicate how many records were excluded by a human and how many were excluded by automation tools.

Source: Page MJ, et al. BMJ 2021;372:n71. doi: 10.1136/bmj.n71.

This work is licensed under CC BY 4.0. To view a copy of this license, visit <https://creativecommons.org/licenses/by/4.0/>
