# Supplementary material for: Treatment Trade‐Offs and Choices for Femoral Fractures: A Systematic Review and Meta‐Analysis
Source: Orthop Surg. 2025 Mar 25;17(5):1298–313. doi: 10.1111/os.70001 (PMC12050177; doi:10.1111/os.70001)
Supplement: Supplementary file 3 — Data S3. Specific Search. [file OS-17-1298-s001.doc]

The specific search strategy is as follows:

1. Search Platforms: Searches were conducted using the PubMed, Web of Science, and Cochrane Library databases.

2. Time Frame: The search was limited to the period from 2015 to 2024 to capture research findings from the past decade.

3. Type of Literature: Abstracts and case reports were excluded, and only original research articles were retrieved.

4. Keyword Combination: The following keyword combinations were used for the search with an "OR" logical relationship:

- Group 1 Keywords: Femoral fracture traction surgery

- This yielded 159 results on PubMed.


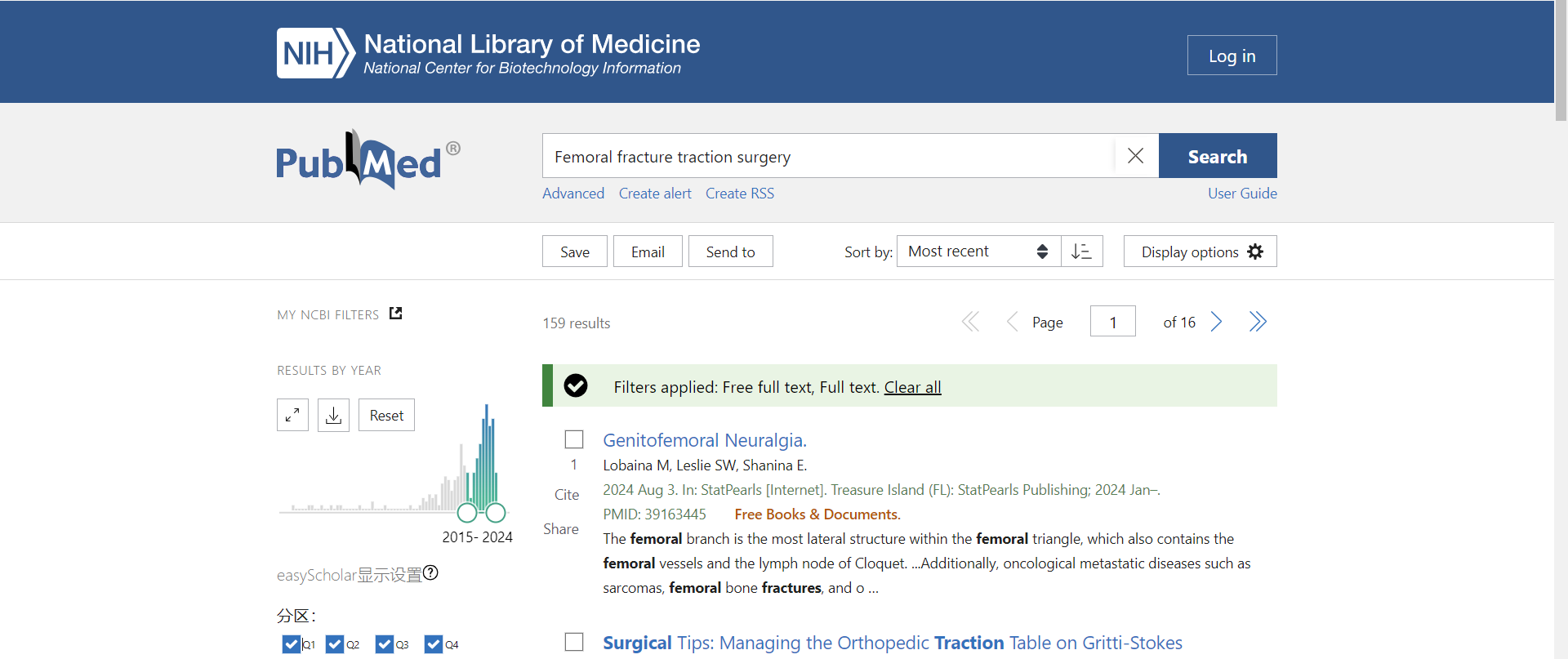


On Web of Science, the search for the keyword combination "Femoral fracture traction surgery" yielded 120 results.


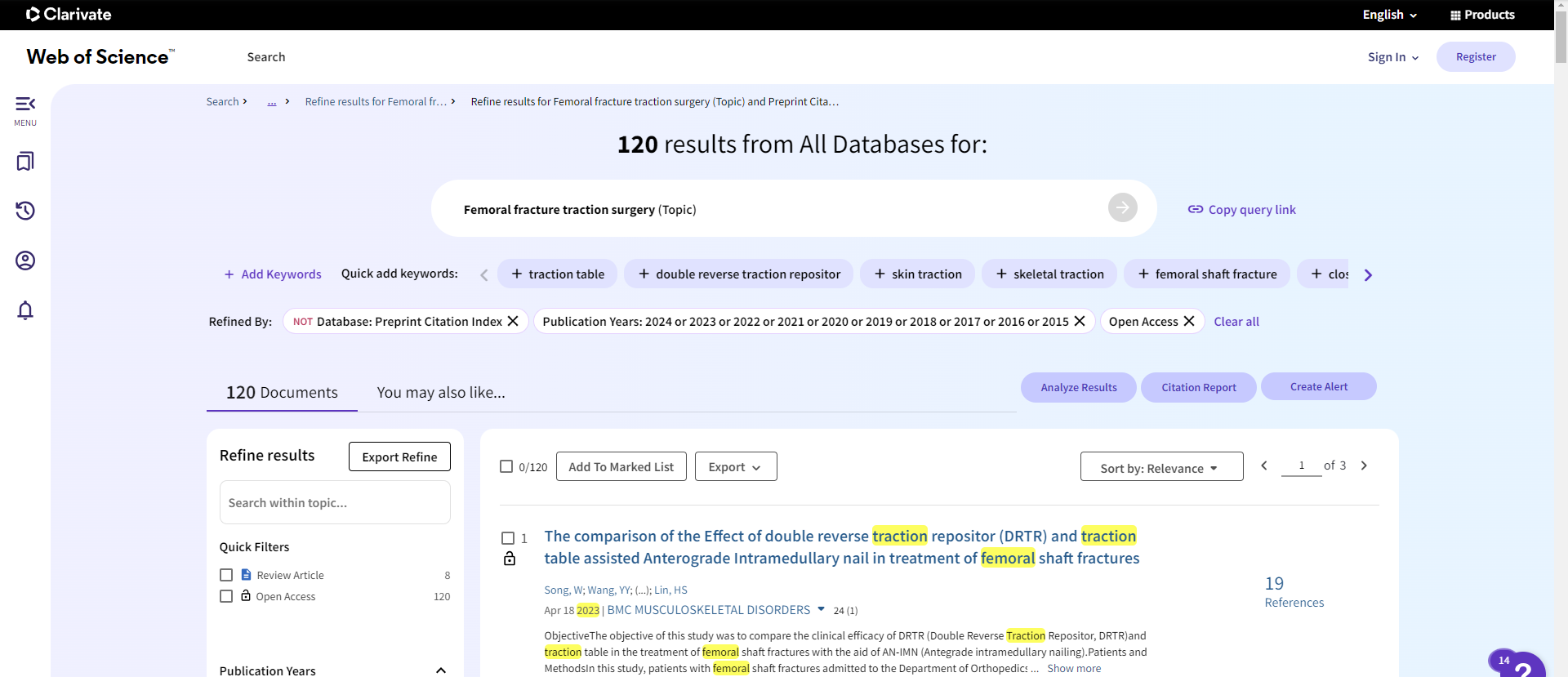


In the Cochrane Library, a search retrieved 68 results.


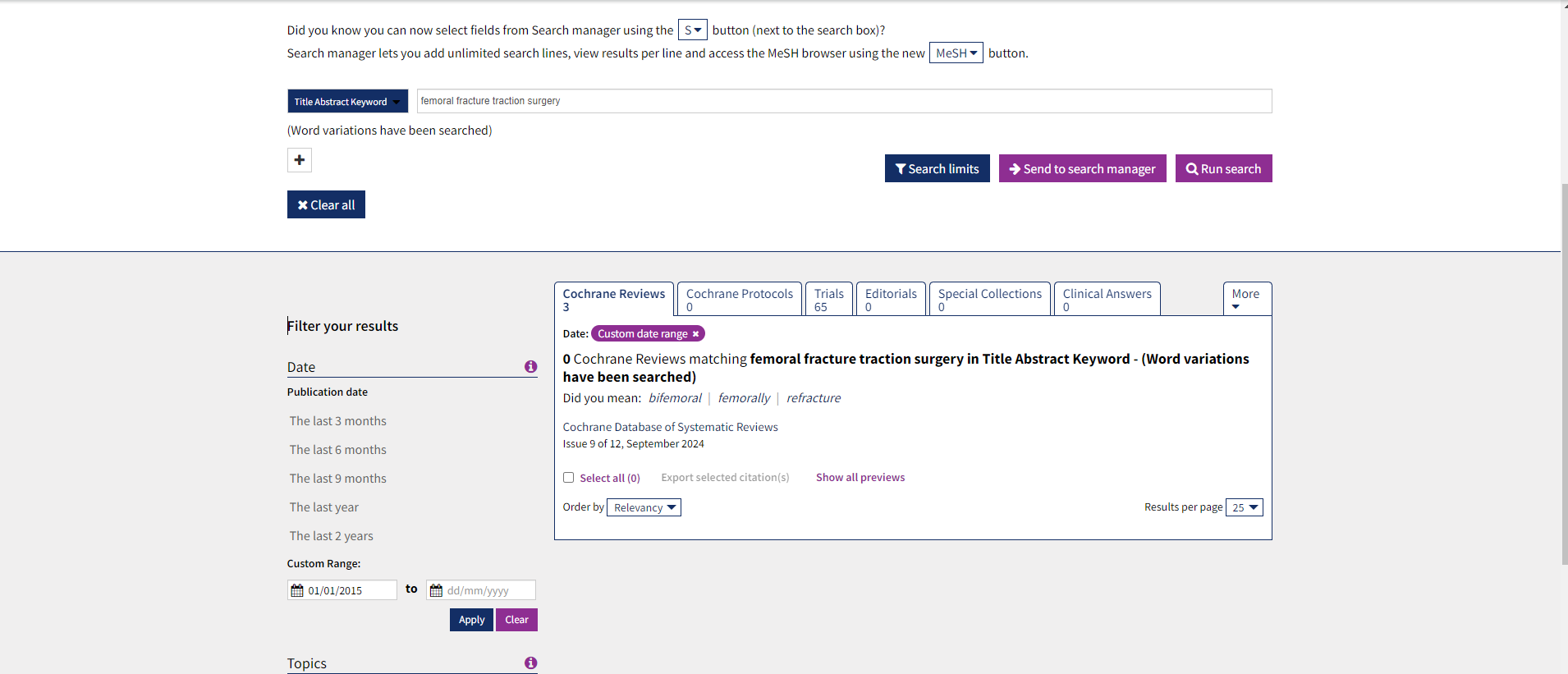


- For the second set of keywords: "Femoral fracture traction surgery efficacy"

- A search on PubMed yielded 11 results.


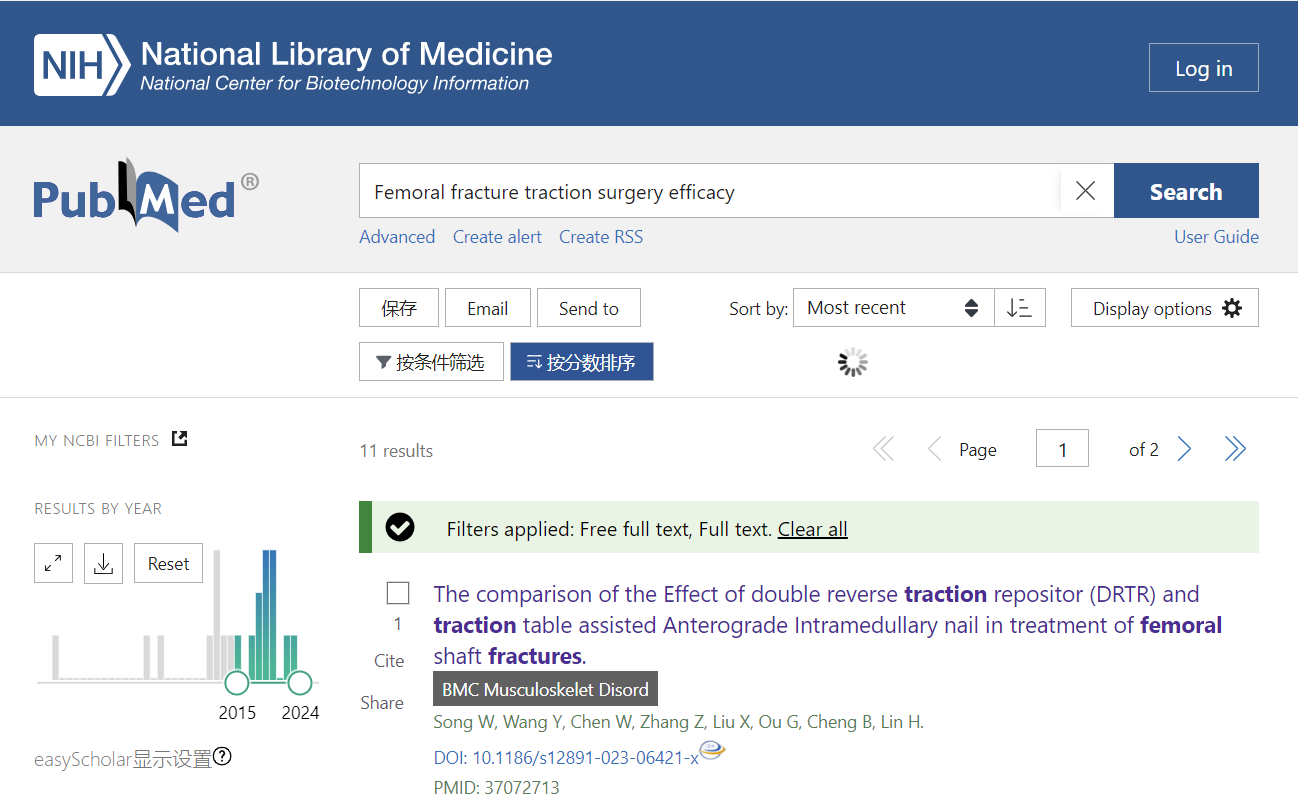


- For the second set of keywords: "Femoral fracture traction surgery efficacy"

- A search on Web of Science yielded 10 results.


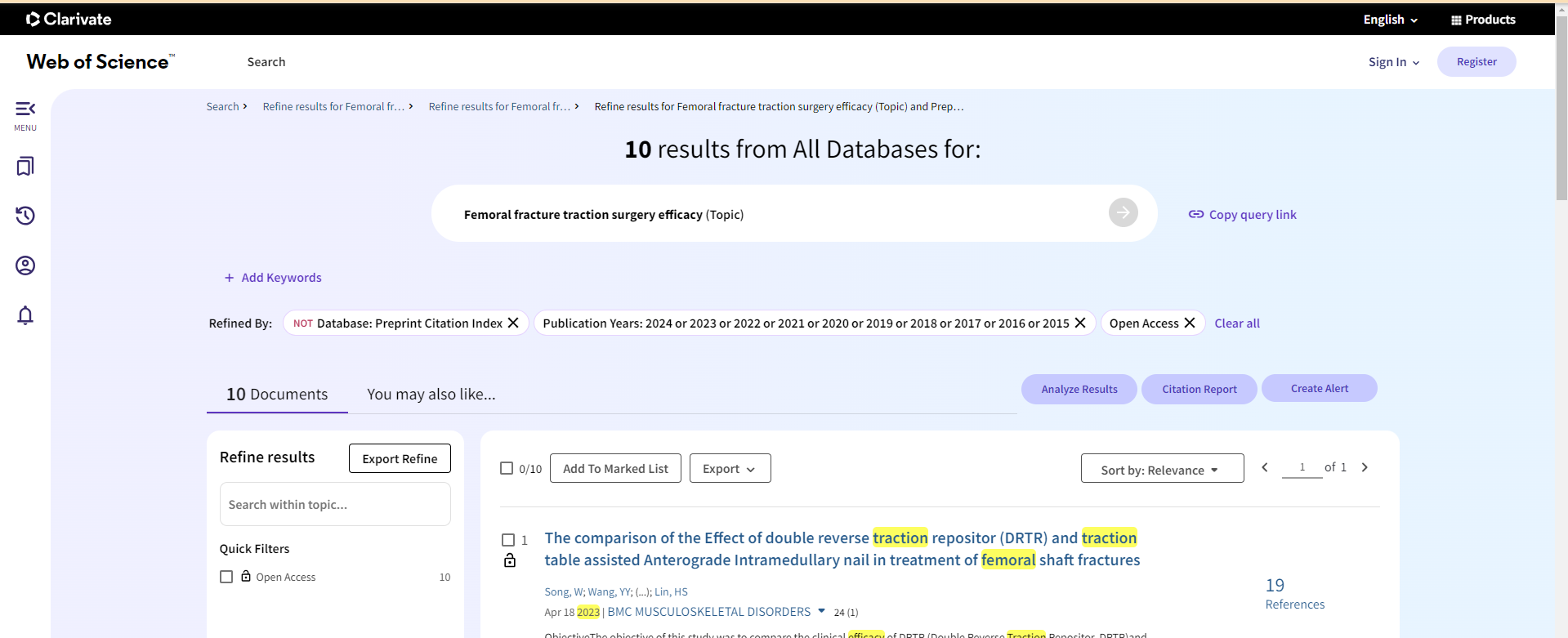


- For the specific query on Cochrane Library, we found 10 relevant results.


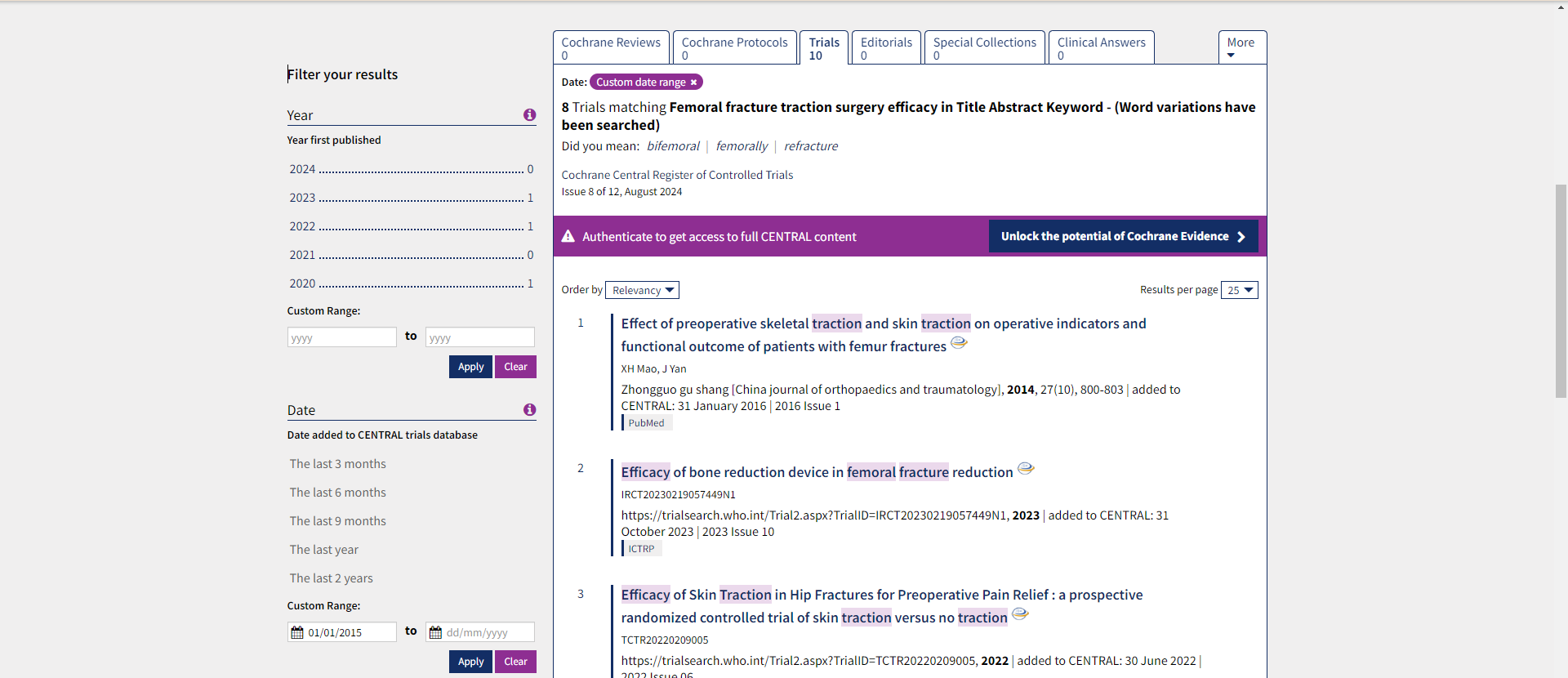


- For the third set of keywords: "Femoral fracture traction surgery cost"

- The search on PubMed yielded 7 results.


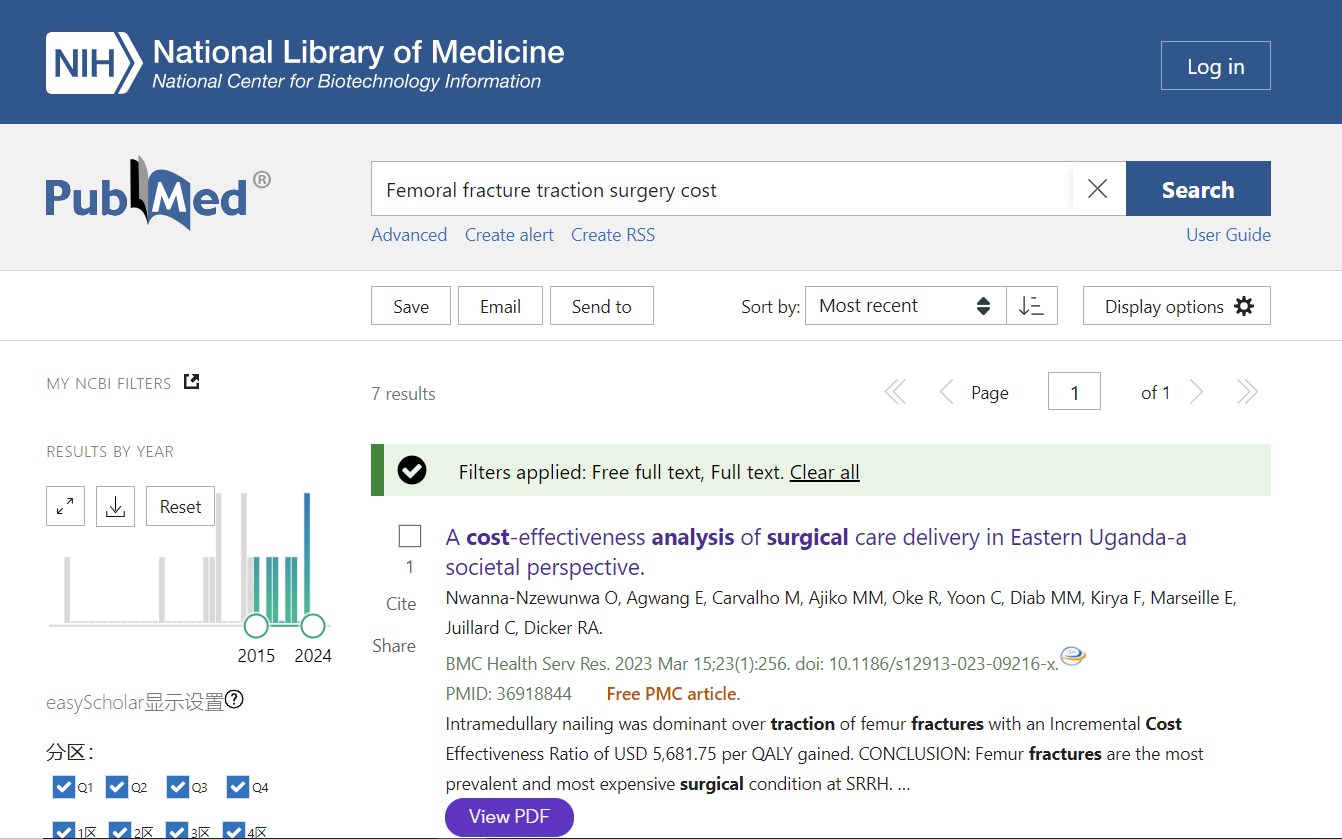


- For the third set of keywords: "Femoral fracture traction surgery cost"

- The search on Web of Science yielded 13 results.


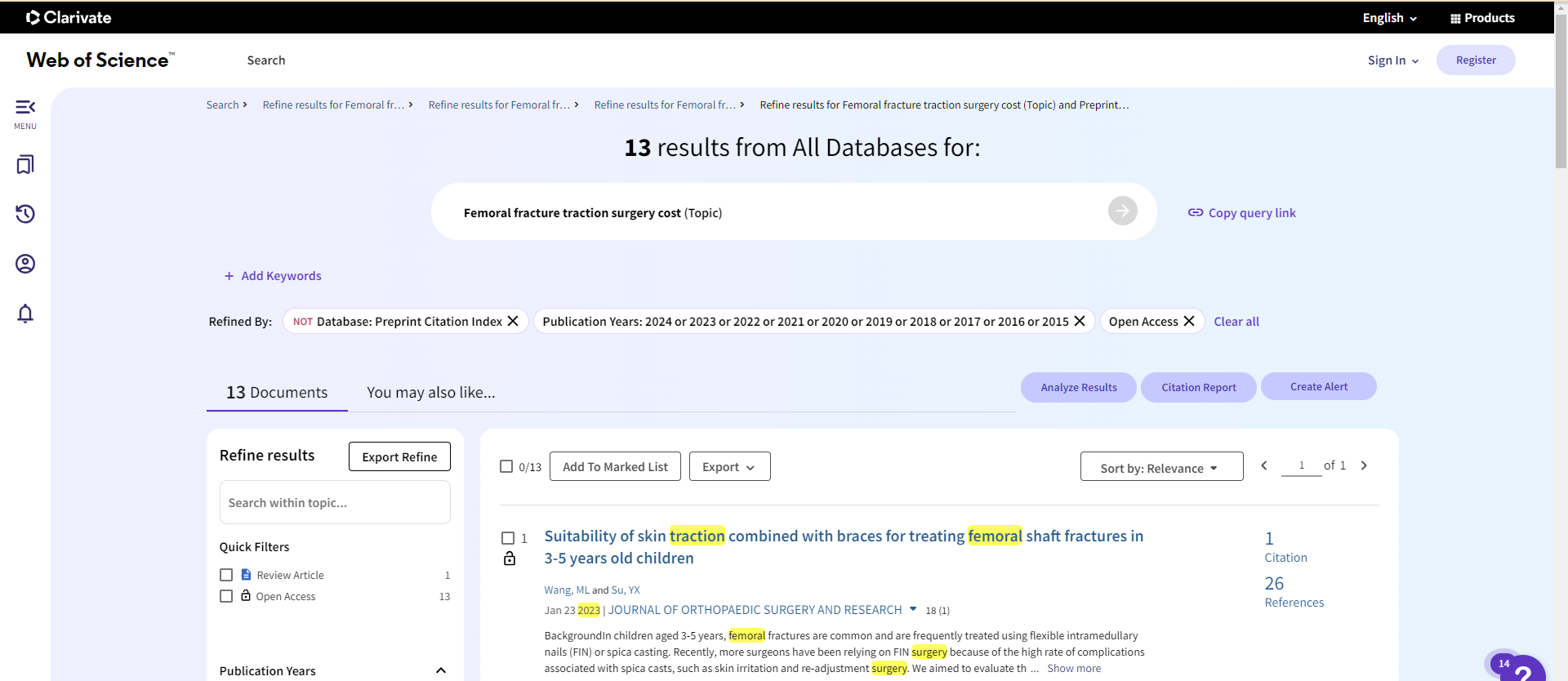


- On Cochrane Library, the search produced 10 results.


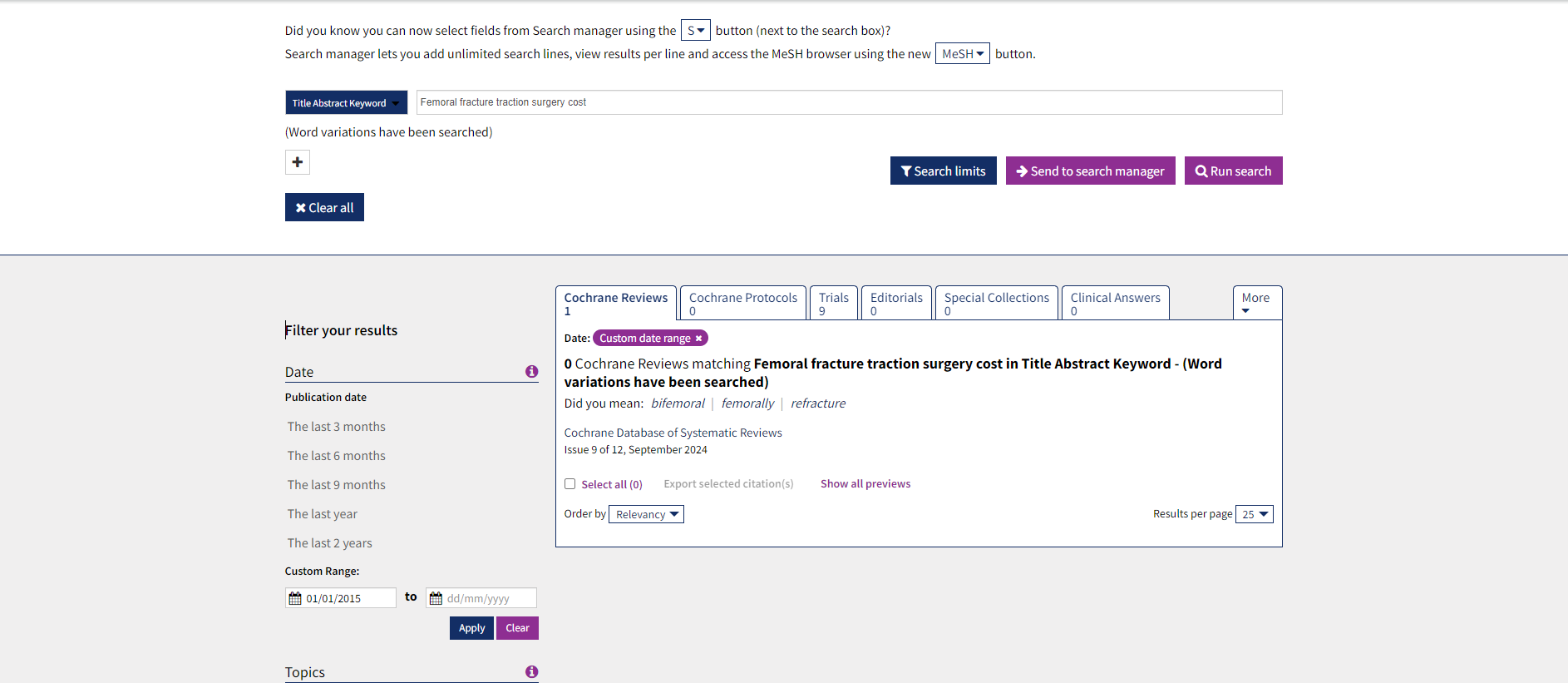


- For the fourth group of keywords: "Femoral fracture traction surgery complications"

- A search on PubMed yielded 83 results.


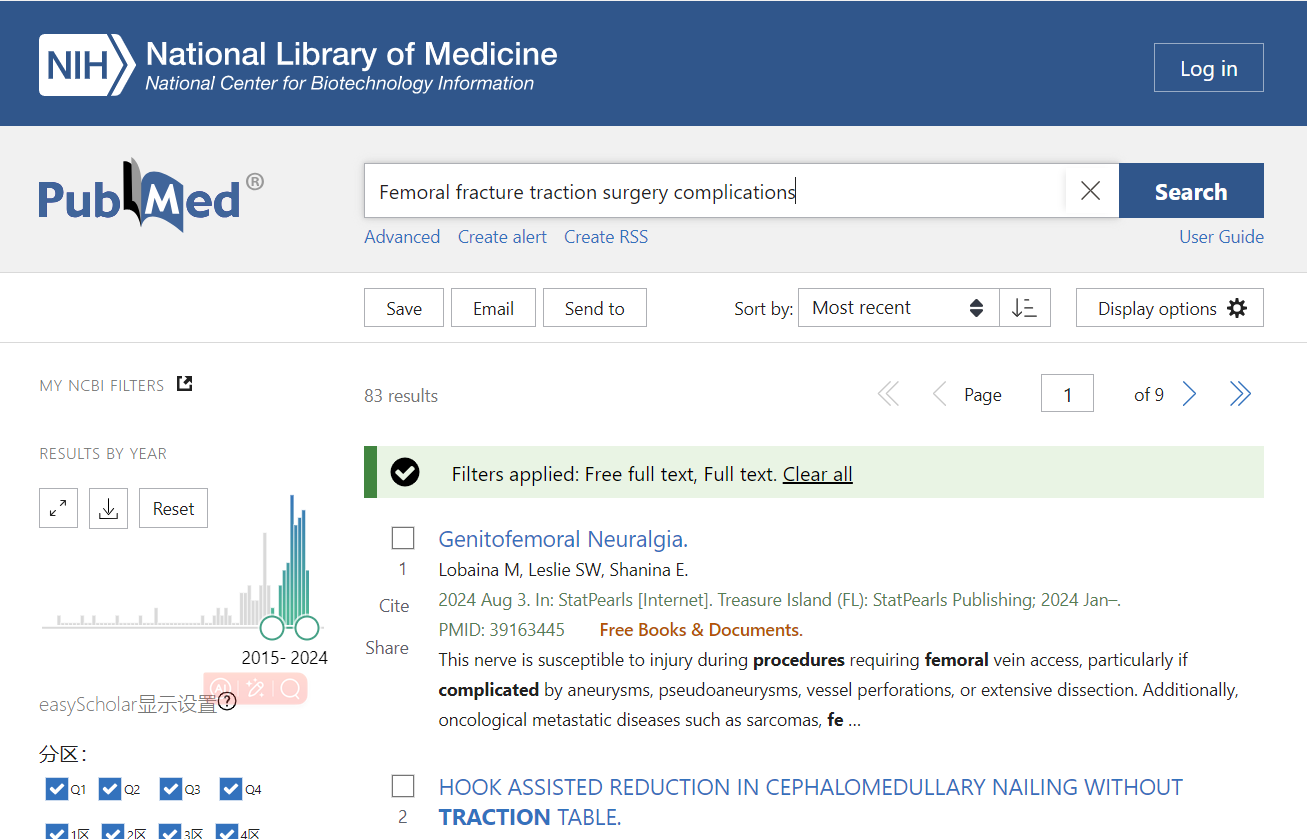


- On Web of Science, the search for "Femoral fracture traction surgery complications" returned 61 results.


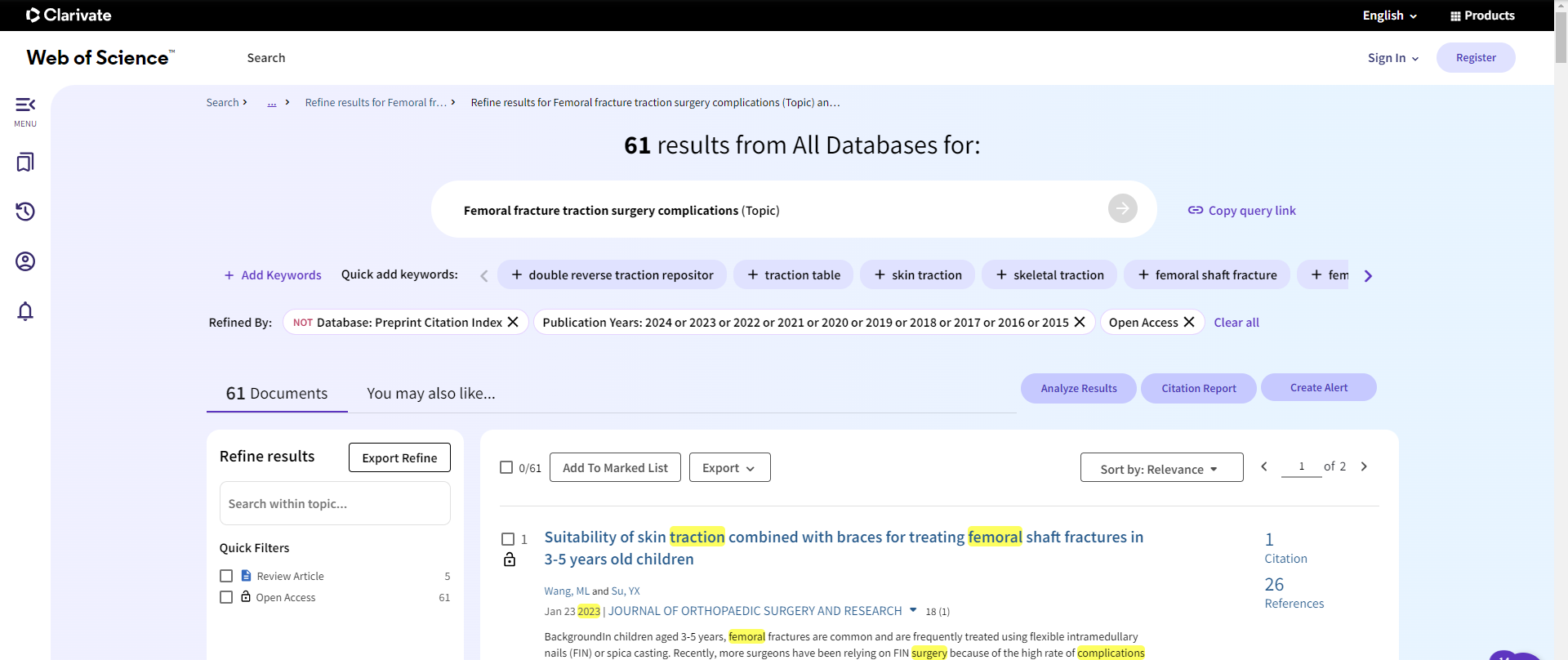


- On the Cochrane Library, the search yielded 10 results.


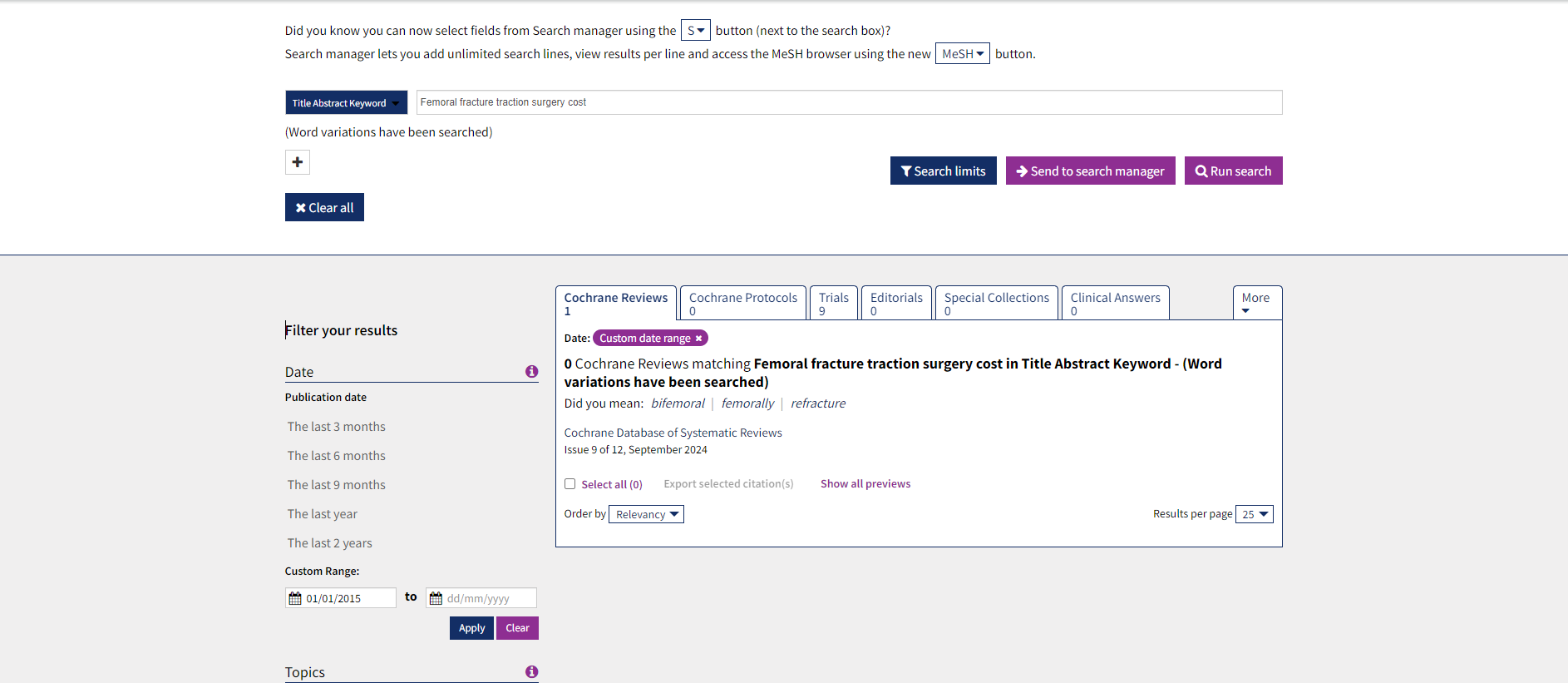


- For the fifth group of keywords: "Femoral fracture traction surgery age"

- A search on PubMed yielded 64 results.


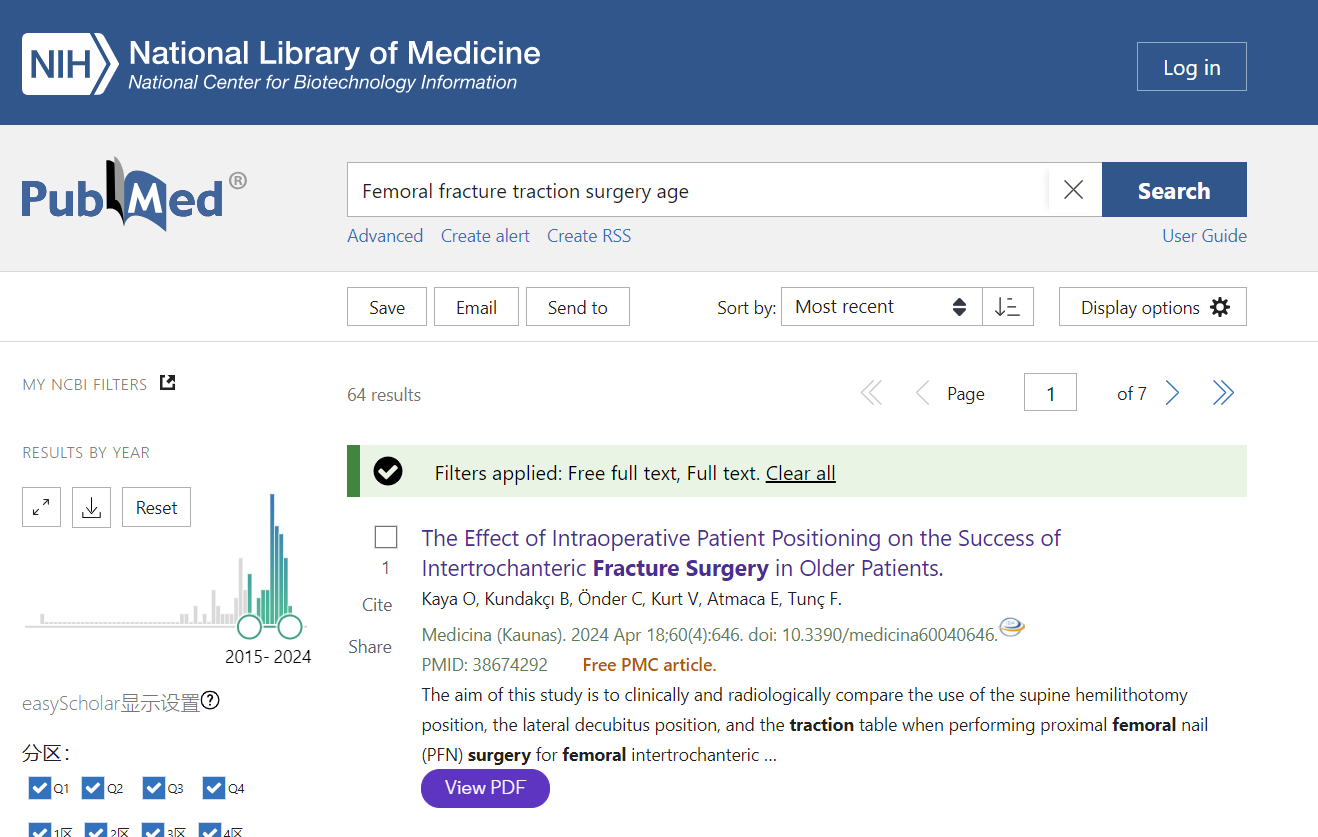


- For the fifth group of keywords: "Femoral fracture traction surgery age"

- A search on Web of Science yielded 66 results.


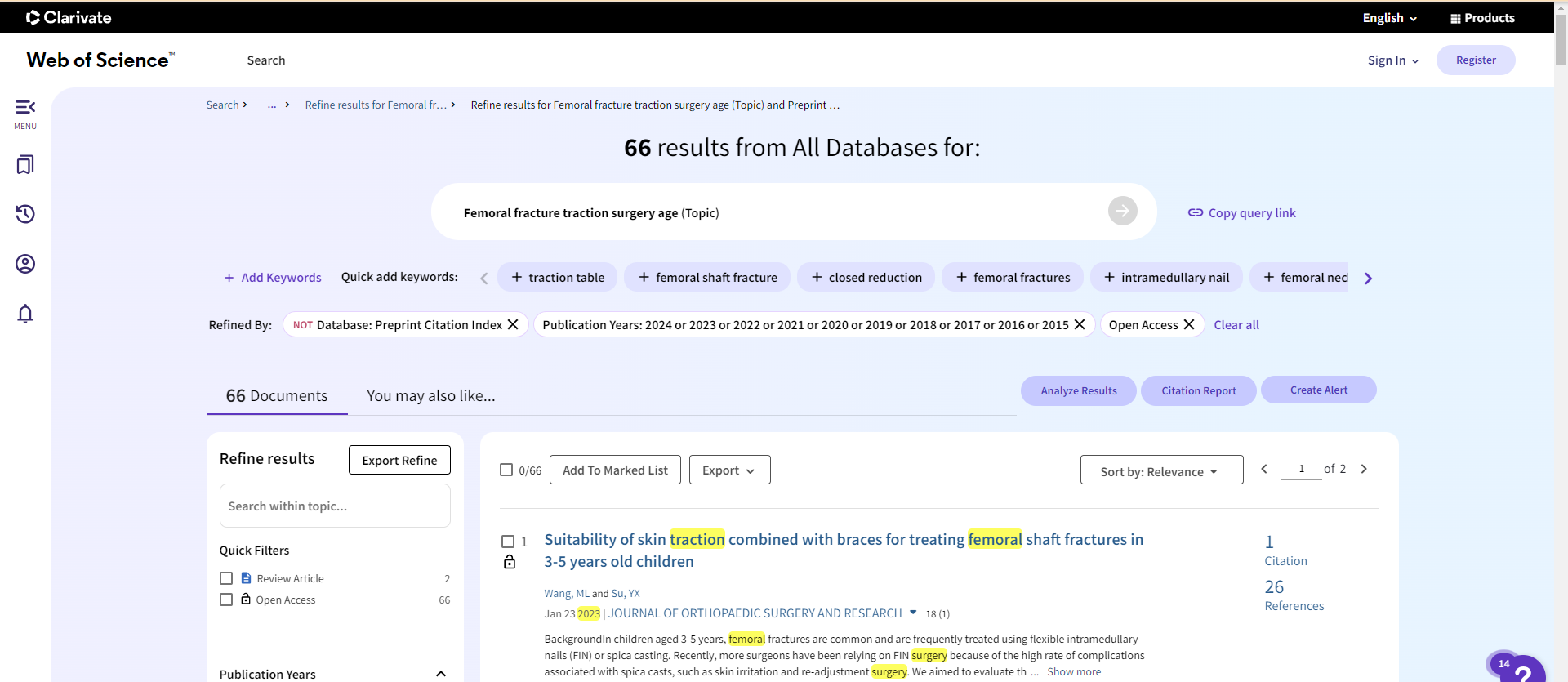


- On the Cochrane Library, a search produced 46 results.


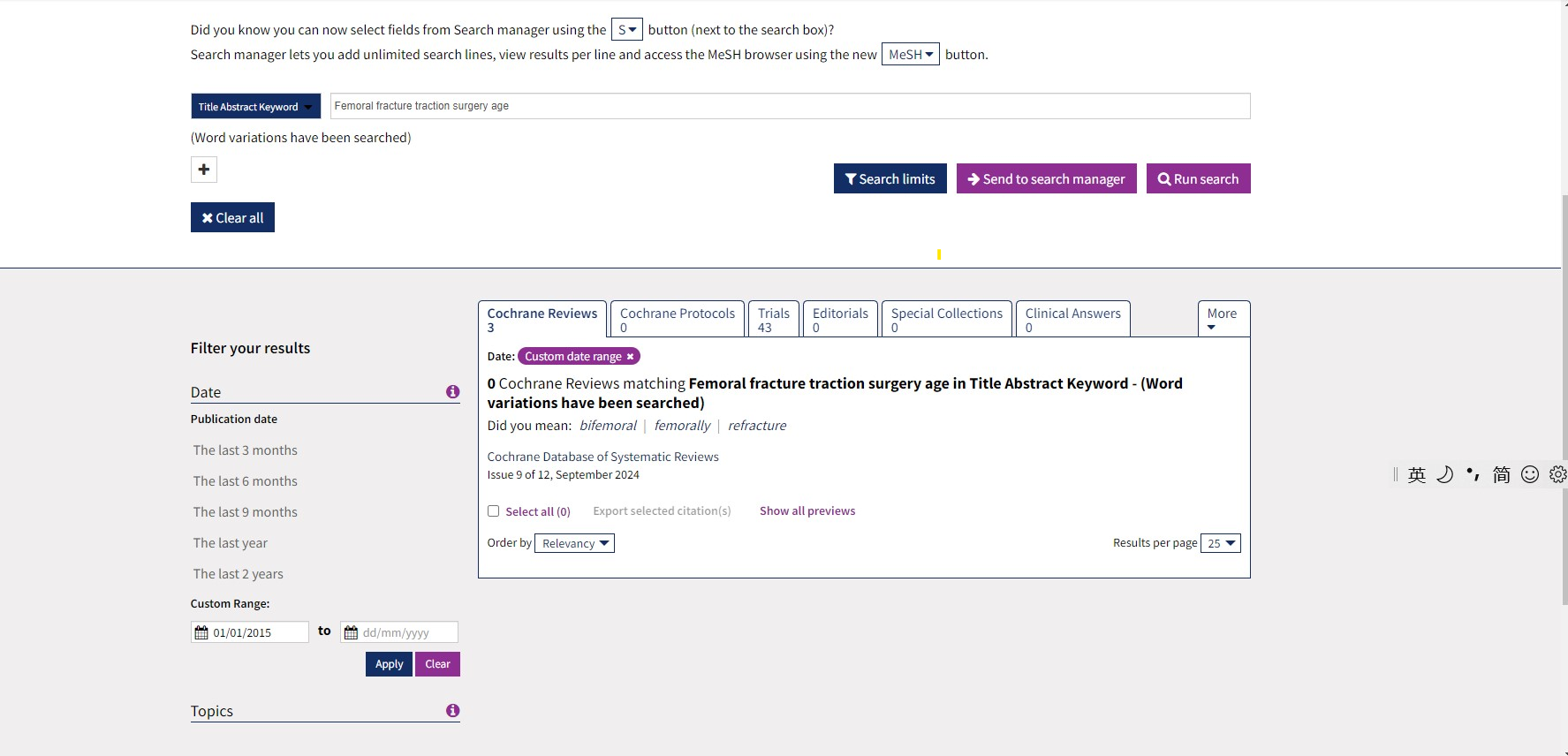


- For the sixth group of keywords: "Femoral fracture traction surgery health"

- A search on PubMed yielded 25 results.


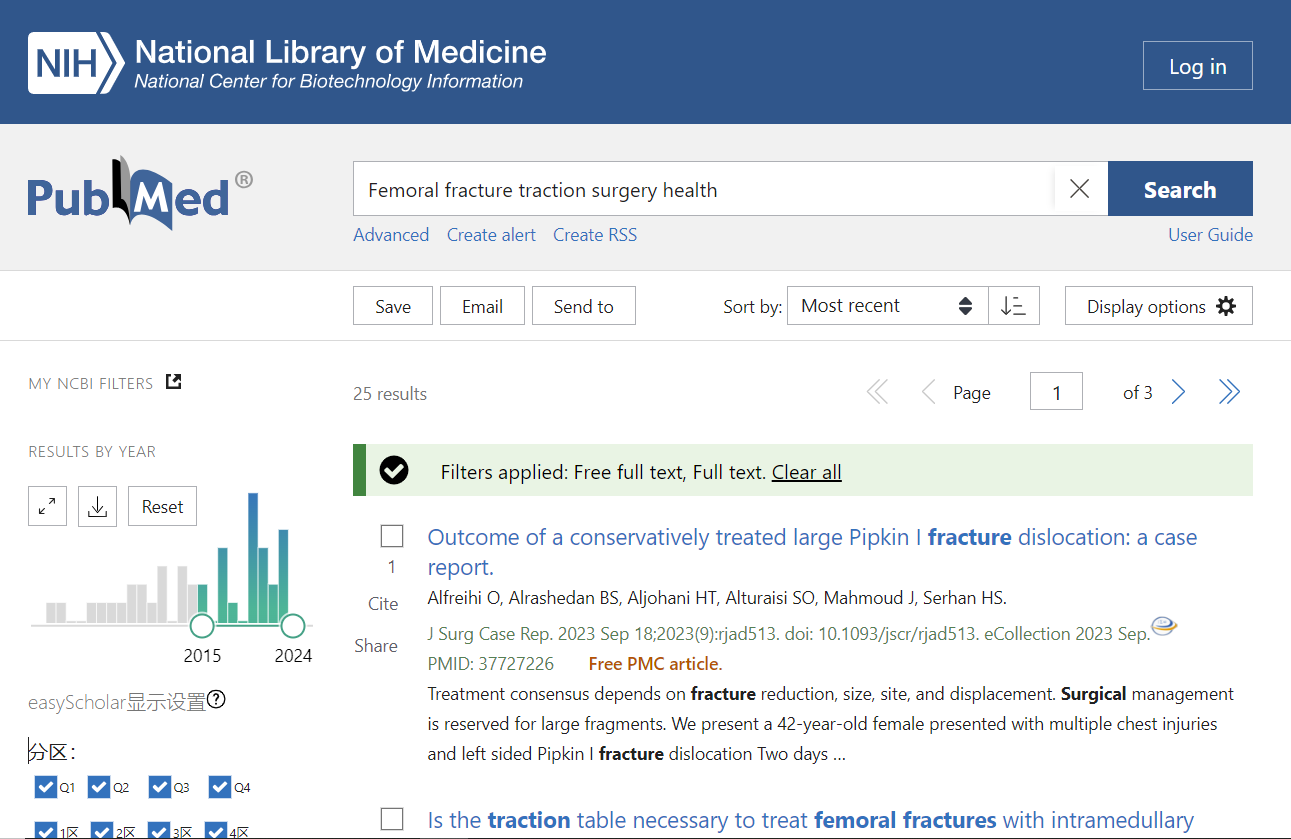


- For the sixth group of keywords: "Femoral fracture traction surgery health"

- A search on Web of Science yielded 14 results.


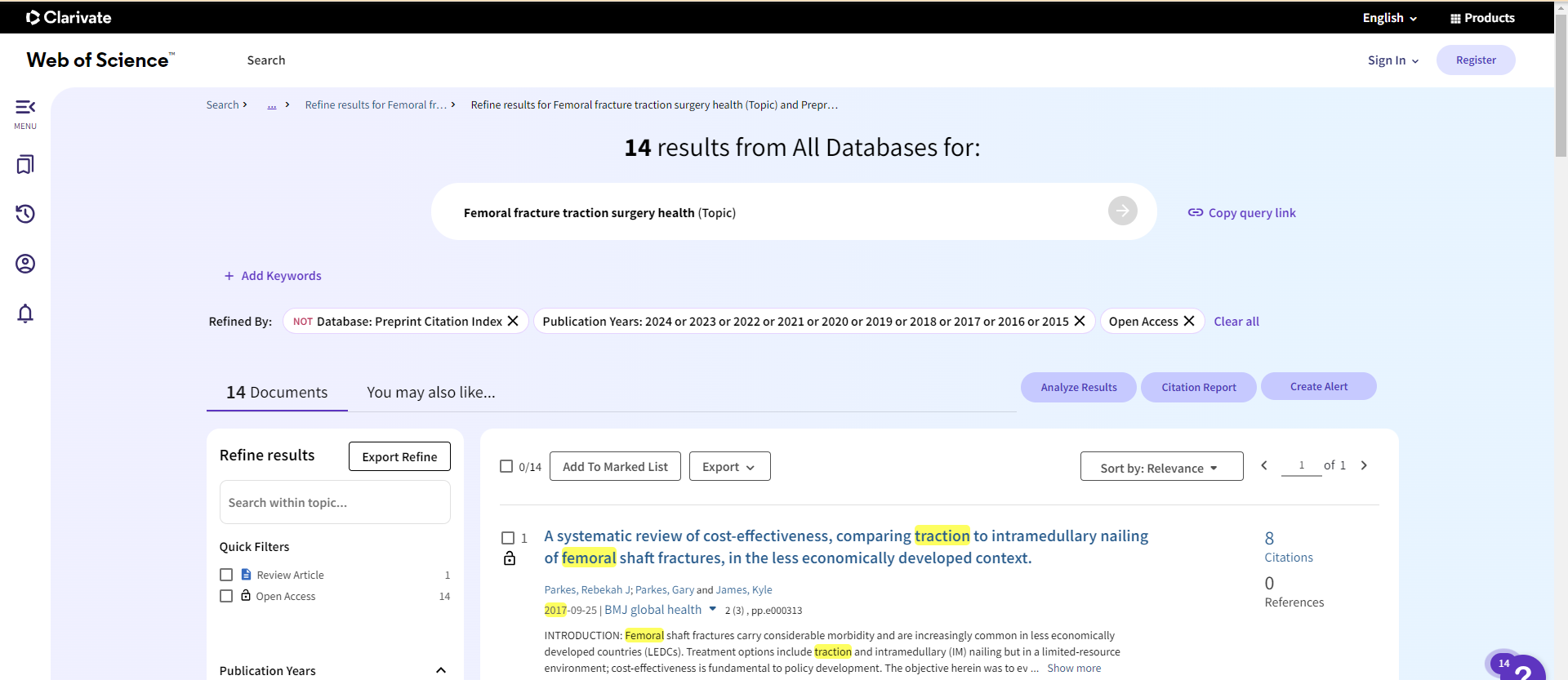


- On the Cochrane Library, a search produced 8 results.


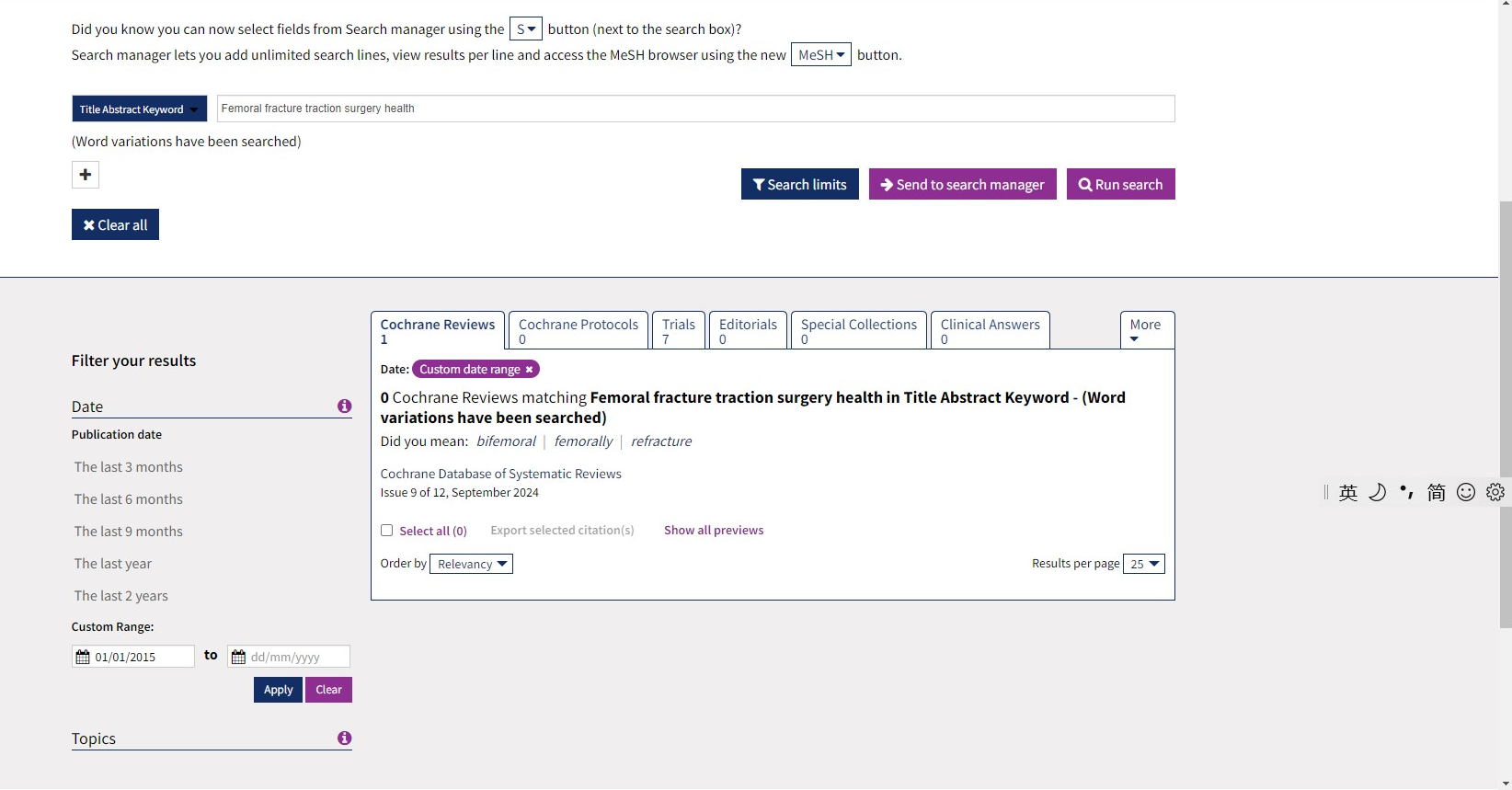


- For the 7th group of keywords: "Femoral fracture traction surgery medical resources"

- The search on PubMed yielded 6 results.


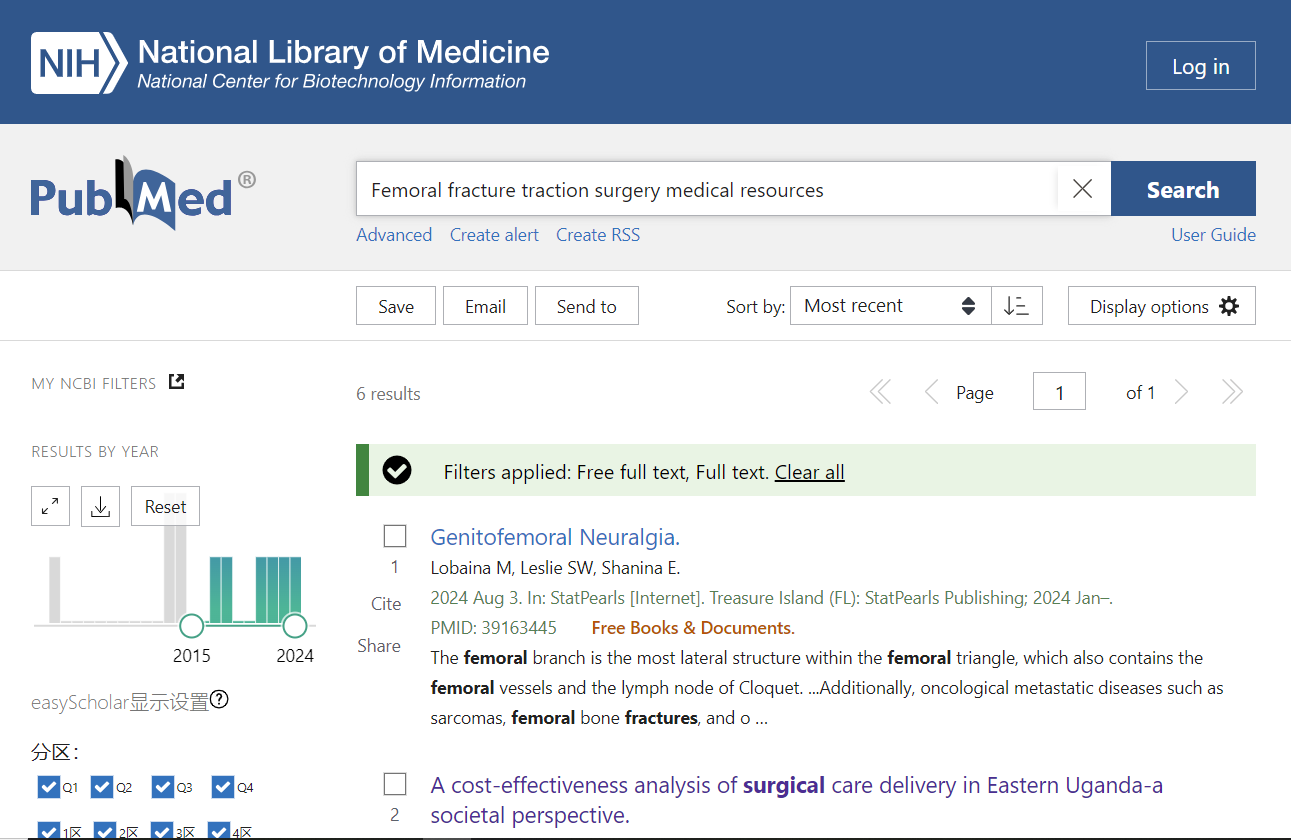


- For the 7th group of keywords: "Femoral fracture traction surgery medical resources"

- The search on Web of Science yielded 4 results.


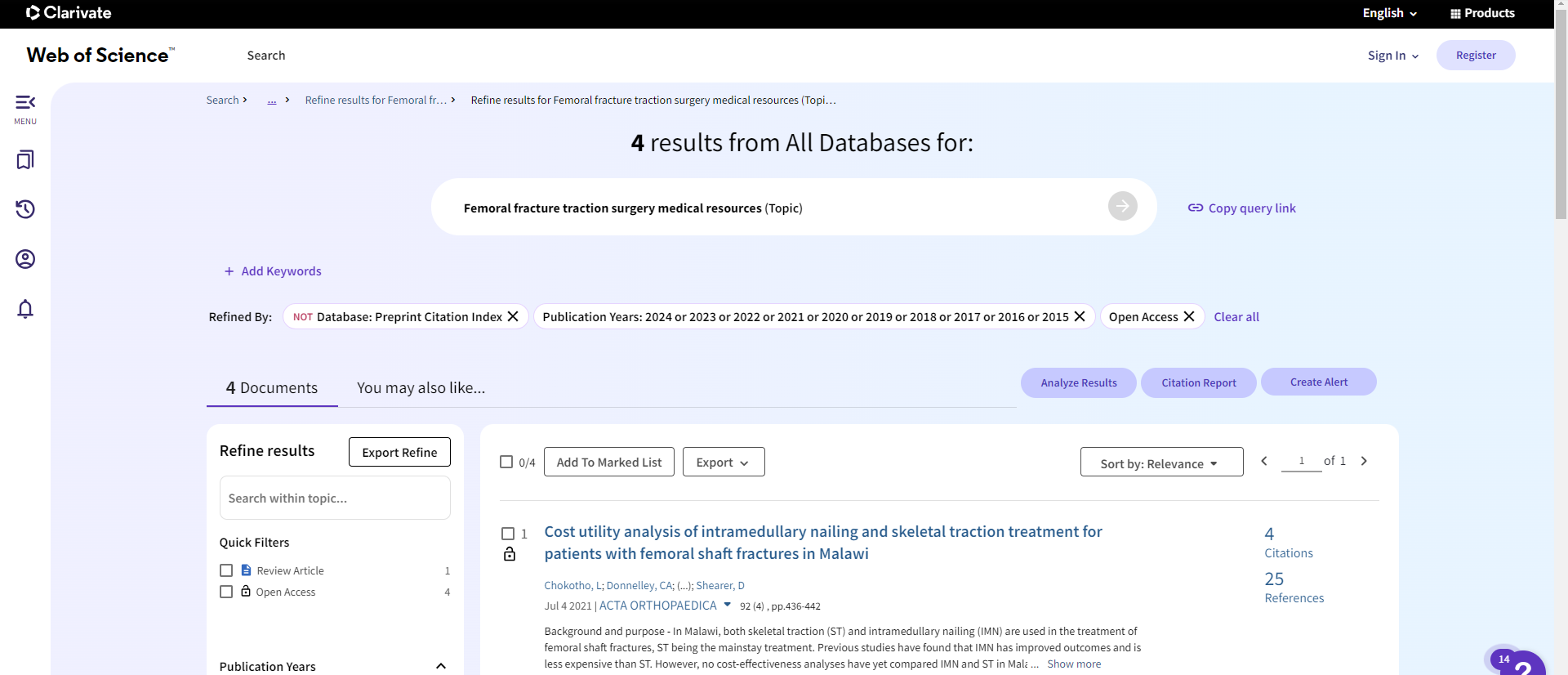


- For the specified search terms, a query on the Cochrane Library produced 1 result.


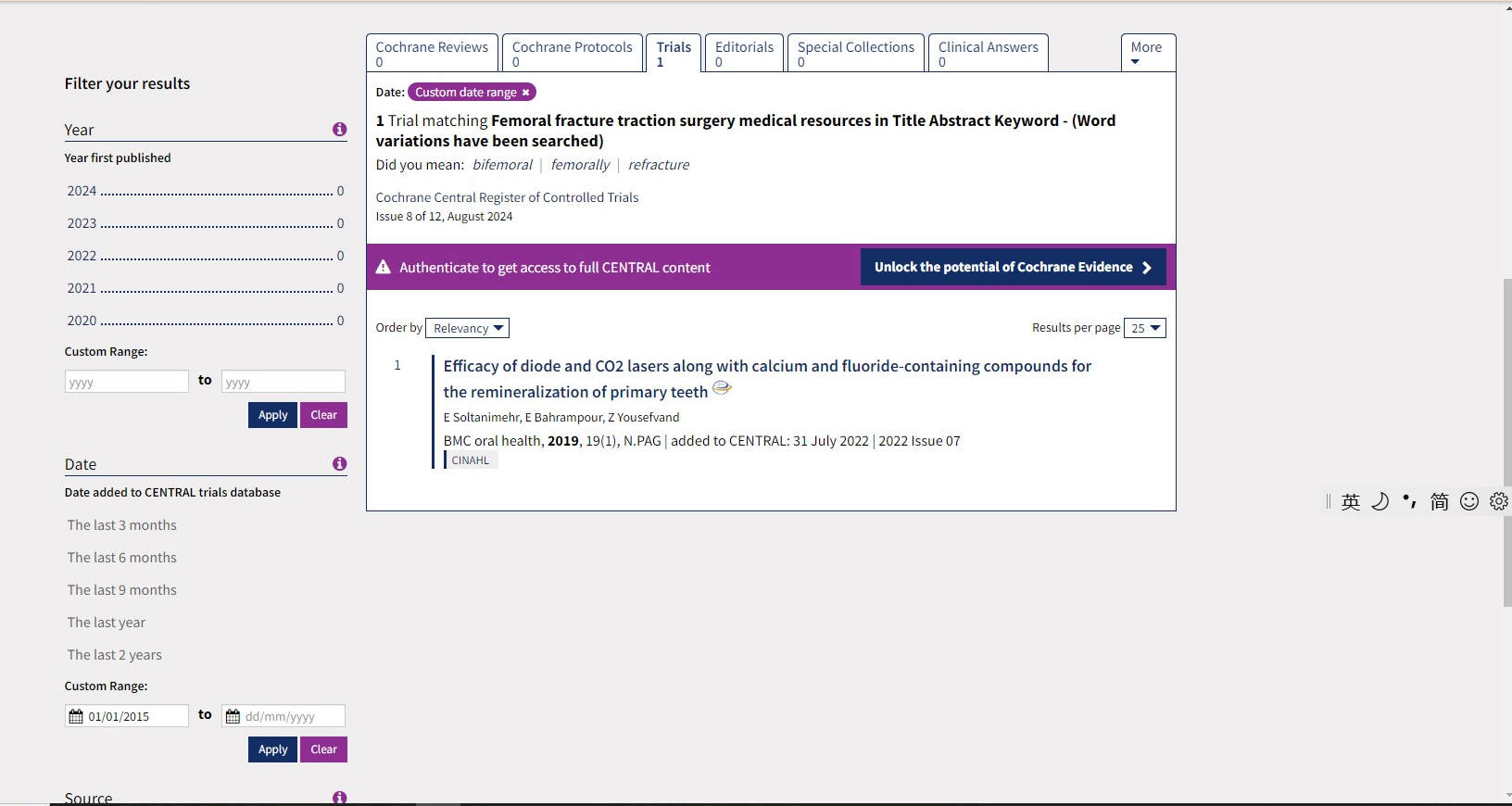


- For the 8th group of keywords: "Femoral fracture traction surgery economy," the search on PubMed yielded 4 results.


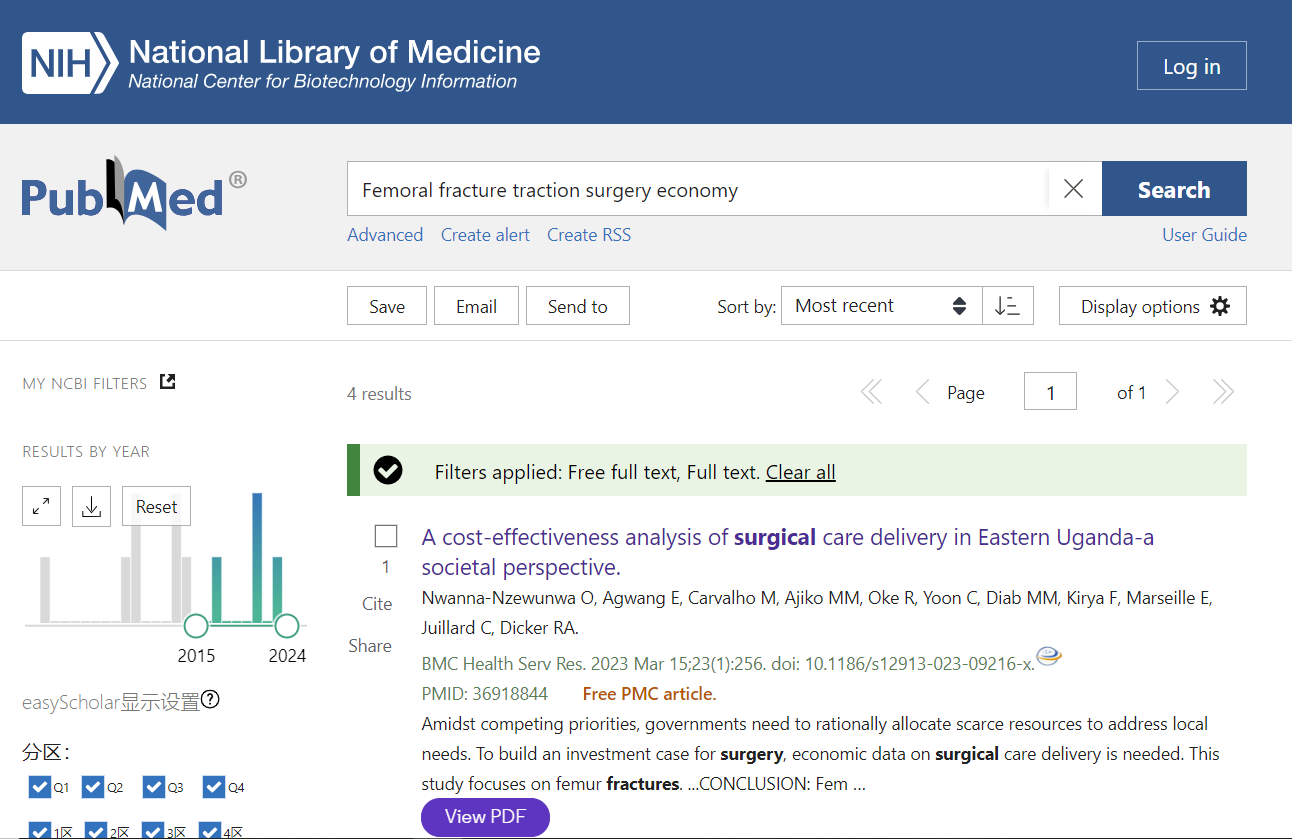


- For the 8th group of keywords: "Femoral fracture traction surgery economy," the search on Web of Science yielded 1 result.


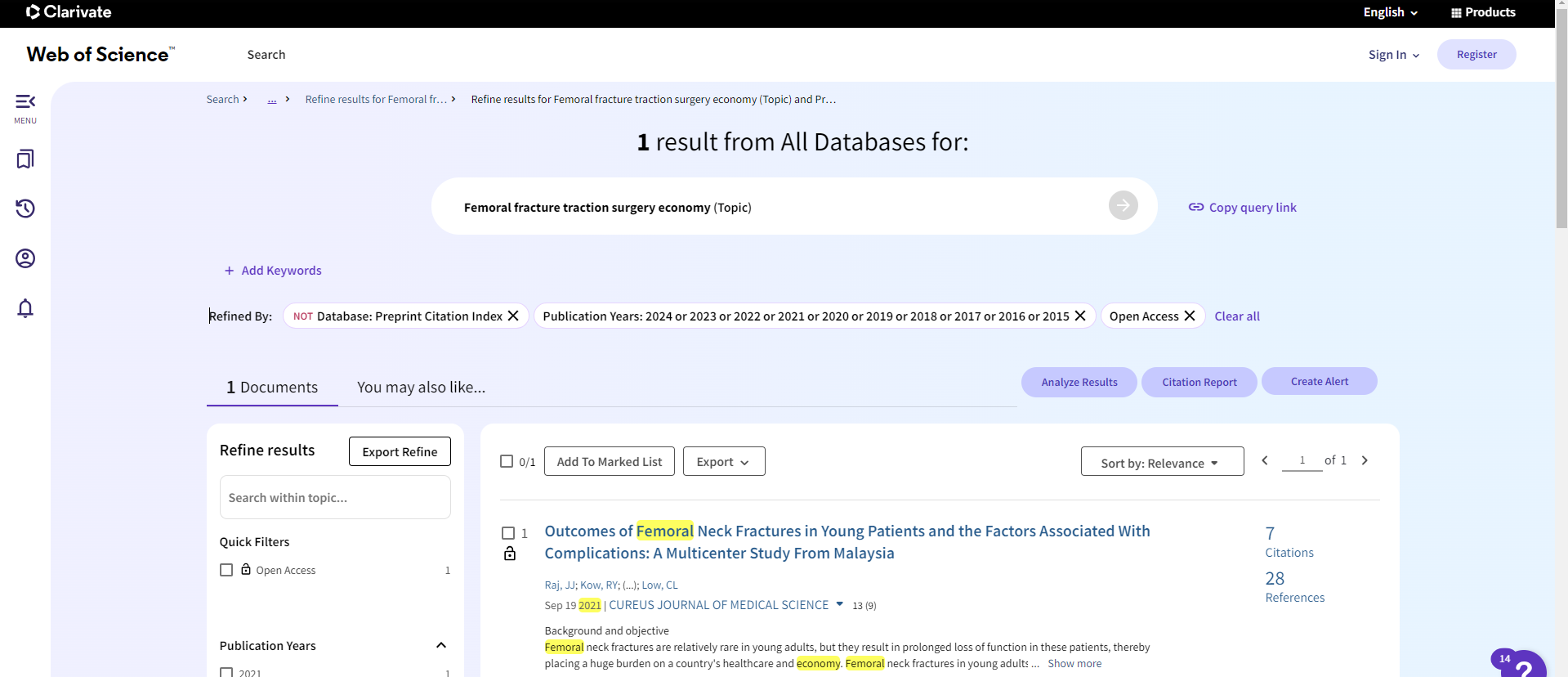


- For the 9th set of keywords: "Femoral fracture traction surgery personal preference," the search on PubMed yielded 4 results.


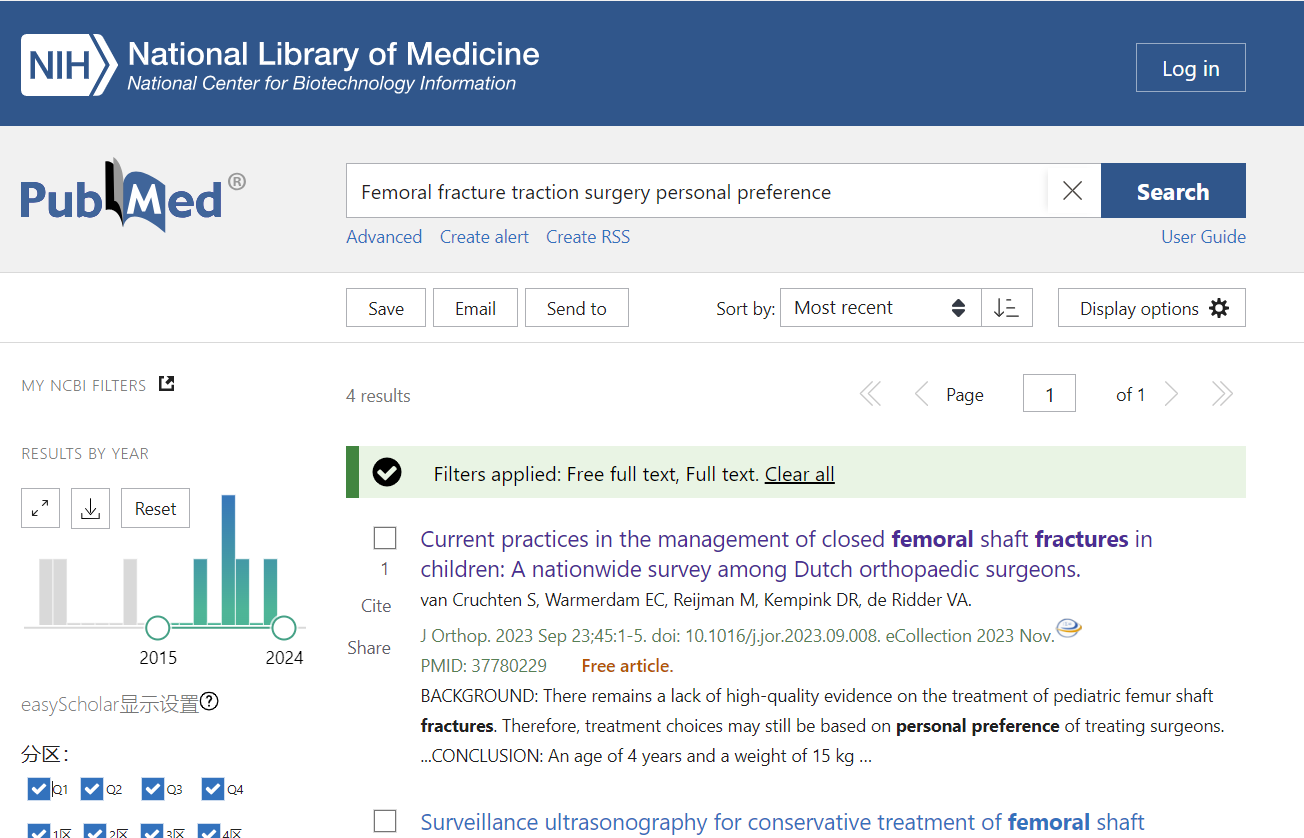


- Retrieved 1 result on Web of Science


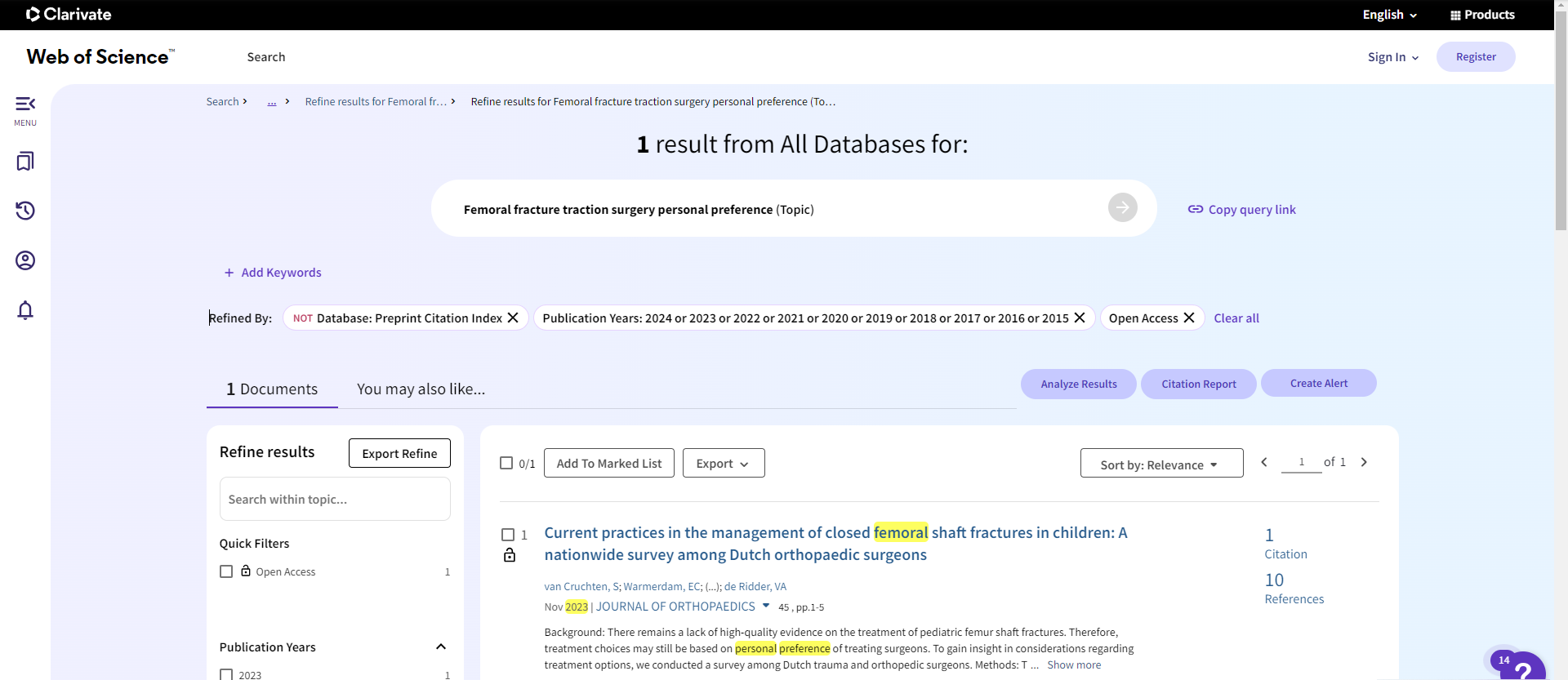


In total: 797 articles

Through the above search strategy, multiple research literatures related to femur fracture traction surgery across various aspects have been retrieved, laying the foundation for further analysis and research.
